# Tumor-intrinsic PIK3CA represses tumor immunogenicity in a model of pancreatic cancer

**DOI:** 10.1172/JCI123540

**Published:** 2019-07-15

**Authors:** Nithya Sivaram, Patrick A. McLaughlin, Han V. Han, Oleksi Petrenko, Ya-Ping Jiang, Lisa M. Ballou, Kien Pham, Chen Liu, Adrianus W.M. van der Velden, Richard Z. Lin

**Affiliations:** 1 Department of Physiology and Biophysics, Stony Brook University, Stony Brook, New York, USA.; 2 Molecular and Cellular Biology Graduate Program, Stony Brook University, Stony Brook, New York, USA.; 3 Department of Molecular Genetics and Microbiology and Center for Infectious Diseases, Stony Brook University, Stony Brook, New York, USA.; 4 Biomedical Engineering Graduate Program, Stony Brook University, Stony Brook, New York, USA.; 5 Department of Pathology and Laboratory Medicine, New Jersey Medical School and Robert Wood Johnson Medical School, Rutgers University School of Medicine, Newark, New Jersey, USA.; 6 Medical Service, Northport VA Medical Center, Northport, New York, USA.

**Keywords:** Oncology, Cancer immunotherapy, Signal transduction, T cells

## Abstract

The presence of tumor-infiltrating T cells is associated with favorable patient outcomes, yet most pancreatic cancers are immunologically silent and resistant to currently available immunotherapies. Here we show using a syngeneic orthotopic implantation model of pancreatic cancer that *Pik3ca* regulates tumor immunogenicity. Genetic silencing of *Pik3ca* in *Kras^G12D^/Trp53^R172H^*-driven pancreatic tumors resulted in infiltration of T cells, complete tumor regression, and 100% survival of immunocompetent host mice. By contrast, *Pik3ca*-null tumors implanted in T cell–deficient mice progressed and killed all of the animals. Adoptive transfer of tumor antigen–experienced T cells eliminated *Pik3ca*-null tumors in immunodeficient mice. Loss of PIK3CA or inhibition of its effector AKT increased the expression of MHC class I and CD80 on tumor cells. These changes contributed to the increased susceptibility of *Pik3ca*-null tumors to T cell surveillance. Our results indicate that tumor cell PIK3CA-AKT signaling limits T cell recognition and clearance of pancreatic cancer cells. Strategies that target this pathway may yield an effective immunotherapy for this cancer.

## Graphical abstract

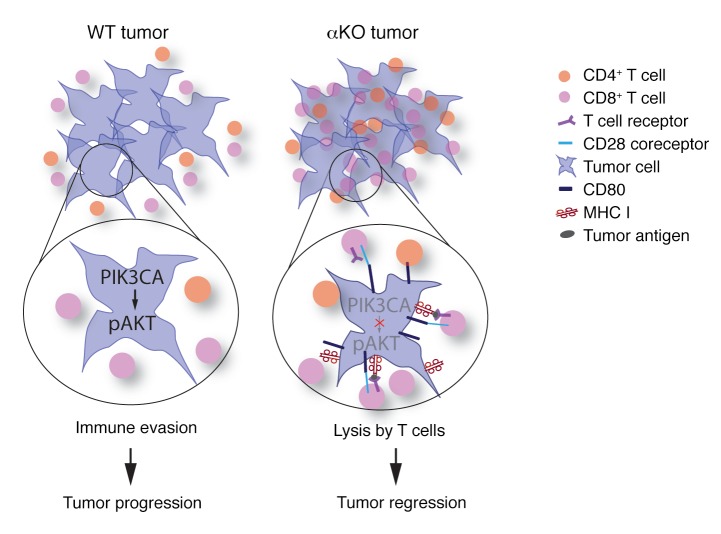

## Introduction

Pancreatic ductal adenocarcinoma (PDAC) is the third leading cause of cancer-related death in the USA, with 44,330 deaths in 2018 and a 5-year survival rate of only 8.5% (1). Standard chemotherapies have little impact on PDAC patient survival, and even those patients who are suitable for surgical resection have only a 10% survival rate past 5 years (2). Presence of tumor-infiltrating T cells is correlated with a better prognosis and increased survival of patients with PDAC (3–6). Recognition of tumor cells by cytotoxic CD8^+^ T cells requires binding of the T cell receptor (TCR) to antigens presented on major histocompatibility complex class I (MHC I), which consists of a membrane-spanning heavy chain and β2 microglobulin (B2m). MHC I is expressed on the surface of all nucleated cells. Pancreatic cancer cell lines isolated from *Kras^G12D^* or *Kras^G12D^ Trp53^R172H^* mice (7) and many human pancreatic cancers and pancreatic cancer cell lines (8, 9) have low MHC I levels that might contribute to immune evasion. In addition to TCR activation, the coreceptor CD28 expressed on T cells is stimulated by CD80. Without CD80, activated T cells become anergic even in the presence of a presented antigen. CD80 is expressed mainly on the surface of professional antigen-presenting cells (APCs) but is sometimes detected at low levels on tumor cells. Upregulation of CD80 on tumor cells has been shown to render them more susceptible to lysis by T cells (10–12).

More than 90% of PDACs have oncogenic mutations in the *KRAS* gene (13). Phosphoinositide-3-kinase (PI3K) is a critical downstream effector of KRAS. Class I PI3Ks are heterodimeric lipid kinases consisting of a regulatory subunit bound to 1 of 4 different p110 catalytic subunits (PIK3CA [also called p110α], PIK3CB, PIK3CD, or PIK3CG). In normal cells, PI3Ks regulate proliferation, survival, and differentiation. Upon activation, PI3Ks catalyze the conversion of phosphatidylinositol-4,5-bisphosphate to phosphatidylinositol-3,4,5-trisphosphate (PIP_3_). PIP_3_ then recruits downstream effectors such as the protein kinase AKT to the cell membrane, aiding in its phosphorylation and activation (14). The PI3K-AKT signaling cascade is among the most frequently dysregulated and hence extensively studied pathways in human cancers (15–17).

PI3Ks in pancreatic cells and in immune cells have been shown to affect pancreatic tumorigenesis and cancer growth. Oncogenic KRAS^G12D^ signals through PIK3CA, but not PIK3CB, to induce acinar-to-ductal metaplasia that is required for pancreatic tumor formation (18), and the catalytic activity of PIK3CA is required for this tumorigenic process (19). It is not yet known if PIK3CA is also required for the growth and progression of established PDAC in vivo. Moreover, PIK3CA’s role in cancer immune surveillance has not been studied. Signaling by PIK3CG and PIK3CD in leukocytes, but not in tumor cells, can affect how the immune system responds to tumors in murine models, including pancreatic cancer (20, 21). Small molecules that inhibit all PI3K isoforms have little effect or transient suppressive effects on PDAC growth in mouse models (22, 23). However, these drugs inhibit PI3Ks in both the tumor cells and immune cells, so the results of systemic inhibitor studies should be interpreted with caution.

In this study, we examined the function of PIK3CA in a pancreatic cancer cell line derived from mice expressing KRAS^G12D^ and TRP53^R172H^ (24, 25). We show that cells lacking PIK3CA are viable in vitro but are cleared by tumor-infiltrating T cells when implanted in the pancreas of immunocompetent mice. The susceptibility of these tumors to immunosurveillance is due at least in part to increased expression of MHC I and CD80 on the tumor cell surface.

## Results

### Pik3ca in implanted KPC cells promotes tumor progression and lethality.

**Figure 1:**
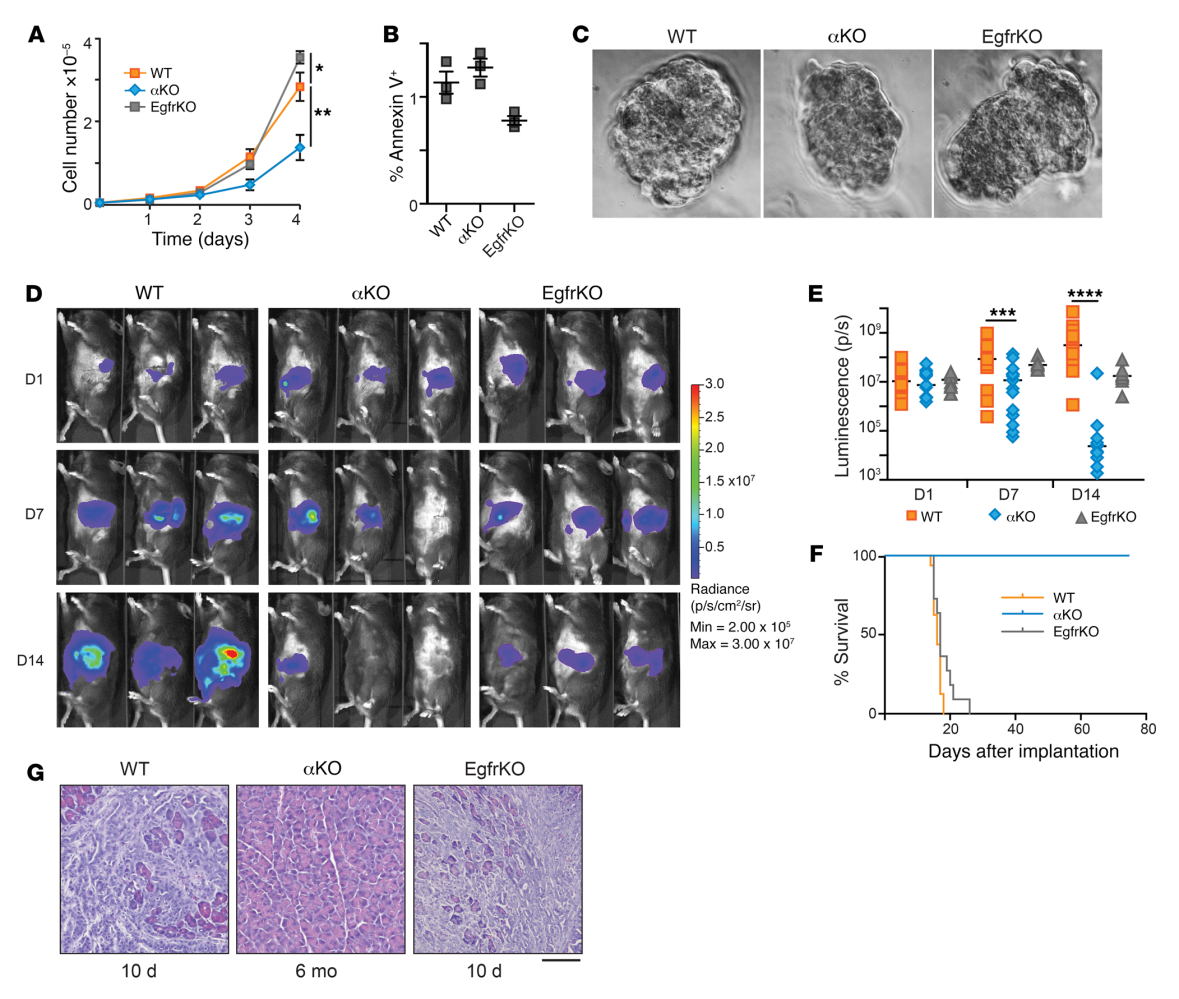
Proliferation of αKO pancreatic cancer cells in vitro and regression in vivo. (**A**) Proliferation rates of KPC cell lines in standard 2D culture. Cells plated in triplicate were counted at the times indicated (mean ± SEM; *n* = 3). *P = 0.0191, WT versus αKO; **P = 0.375, WT versus EgfrKO (1-way ANOVA with Bonferroni’s post hoc test). (**B**) Percentage of cells positive for annexin V staining at the 4-day time point in **A** (mean ± SEM, *n* = 3). *P* = 0.18, WT versus αKO; *P* = 0.12, WT versus EgfrKO (1-way ANOVA with Bonferroni’s post hoc test). (**C**) Representative light microscopy images (×40 magnification) of spheroids formed after 5 days in 3D methylcellulose culture. (**D**) Cells (0.5 million) were implanted in the head of the pancreas of B6 mice. Tumor growth was monitored by IVIS imaging of the luciferase signal on days 1, 7, and 14 after implantation. Representative images of 3 mice in each group are shown. (**E**) Quantification of luciferase signals from each mouse. The bars indicate median. On day 7, ***P = 0.0006, WT versus αKO and *P* > 0.9, WT versus EgfrKO; on day 14, ****P < 0.0001, WT versus αKO and *P* = 0.0618, WT versus EgfrKO (Kruskal-Wallis test with Dunn’s post hoc test). (**F**) Kaplan-Meier survival curves for B6 mice implanted with the indicated cell lines. Median survival: WT, 16 days (*n* = 16); EgfrKO, 17 days (*n* = 10); all αKO mice were alive at day 80 (*n* = 17). *P* < 0.0001, WT versus αKO (log-rank test). (**G**) H&E-stained sections of pancreatic tissue collected at the indicated times after cell implantation in B6 mice. Scale bar: 100 μm.

Both *Pik3ca* and *Egfr* have been shown to be required for KRAS^G12D^-induced pancreatic tumorigenesis (18, 19, 26). To investigate the possible roles of *Pik3ca* and *Egfr* in pancreatic cancer progression, we used the FC1245 pancreatic cancer cell line that was isolated from a *Kras^LSL–G12D/+^ Trp53^LSL–R172H/+^ Pdx1-Cre* mouse in the C57BL/6 (B6) genetic background (25). We first produced a parental cell line that stably expresses luciferase (referred to as WT KPC) and then used CRISPR/Cas9 to produce KPC cell lines that lack either *Pik3ca* or *Egfr* (referred to as αKO or EgfrKO, respectively). Complete loss of PIK3CA or EGFR was confirmed by Western blotting (Supplemental Figure 1A). Immunoblotting and reverse phase protein array (RPPA) analysis also revealed changes in signaling due to ablation of *Pik3ca* or *Egfr*. In particular, there was a large decrease in AKT phosphorylation at S473 and T308 in αKO cells as compared with WT cells, whereas EgfrKO cells had higher levels of AKT phosphorylation than WT cells (Supplemental Figure 1). EgfrKO KPC cells proliferated at a higher rate than WT cells in standard 2D culture conditions, whereas αKO cells proliferated at about half the rate of WT cells (Figure 1A). The percentage of cells stained with annexin V in each culture was not significantly different, indicating that the decreased proliferation rate of αKO KPC cells is not due to increased apoptosis (Figure 1B). When grown under 3D culture conditions, all 3 cell lines formed compact spheroid colonies (Figure 1C), indicating a capacity for anchorage-independent growth.

We next studied the growth of these cell lines in vivo. WT, αΚΟ, or EgfrKO cells were implanted in the head of the pancreas of syngeneic B6 mice, and tumor growth was monitored longitudinally using an IVIS Lumina III in vivo imaging system. As expected, WT cells grew rapidly and formed large tumors (Figure 1D). Median tumor volume quantified as total luminescence flux showed a 29.6-fold increase from day 1 to day 14 (Figure 1E). Tumors formed by EgfrKO cells grew at a slower rate (Figure 1, D and E). Mice implanted with WT or EgfrKO cells died 2 to 3 weeks after implantation, with a median survival of 16 days or 17 days, respectively (Figure 1F and Table 1). In contrast, tumors formed by implanted αKO cells showed an increase in median size from day 1 to day 7, and then the tumors regressed so that the luciferase signal was undetectable in 16 of 17 mice on day 14 (Figure 1, D and E). By day 21, the luciferase signal was undetectable in all 17 mice (data not shown). All B6 mice implanted with αKO cells were alive 80 days later (Figure 1F and Table 1). Some of these convalescent animals have been kept for up to 18 months after tumor implantation without any overt signs of illness. Implantation of B6 mice with another clone of αKO cells yielded similar results (Supplemental Figure 2). At necropsy, mice implanted with WT or EgfrKO cells exhibited large pancreatic tumors and metastases to the peritoneum, liver, diaphragm, and lungs. H&E staining of pancreatic sections confirmed the presence of large tumors with a strong desmoplastic response (Figure 1G). Some of the mice implanted with αKO cells were euthanized after 6 months. No abnormal lesions were seen by visual inspection in any of the organs, and no tumors were found in pancreatic sections of these animals (Figure 1G).

Increased survival was also seen in mice implanted with a different KPC cell line (DT10022) with genetically downregulated *Pik3ca* (αKD cells; Supplemental Figure 3A). All B6 mice implanted with WT DT10022 cells died from tumor progression by day 35, whereas all mice implanted with αKD cells were still alive at that time (Supplemental Figure 3B). Two of 6 mice in the αKD group had no visible tumors at necropsy, and histological examination revealed that the tumor had completely regressed in one animal (Supplemental Figure 3C). We suspect that the tumor regression phenotype is not as robust as with αKO KPC cells because *Pik3ca* is not completely ablated in the αKD DT10022 cell line.

### Regression of αKO KPC tumors is linked to T cell infiltration.

**Figure 2:**
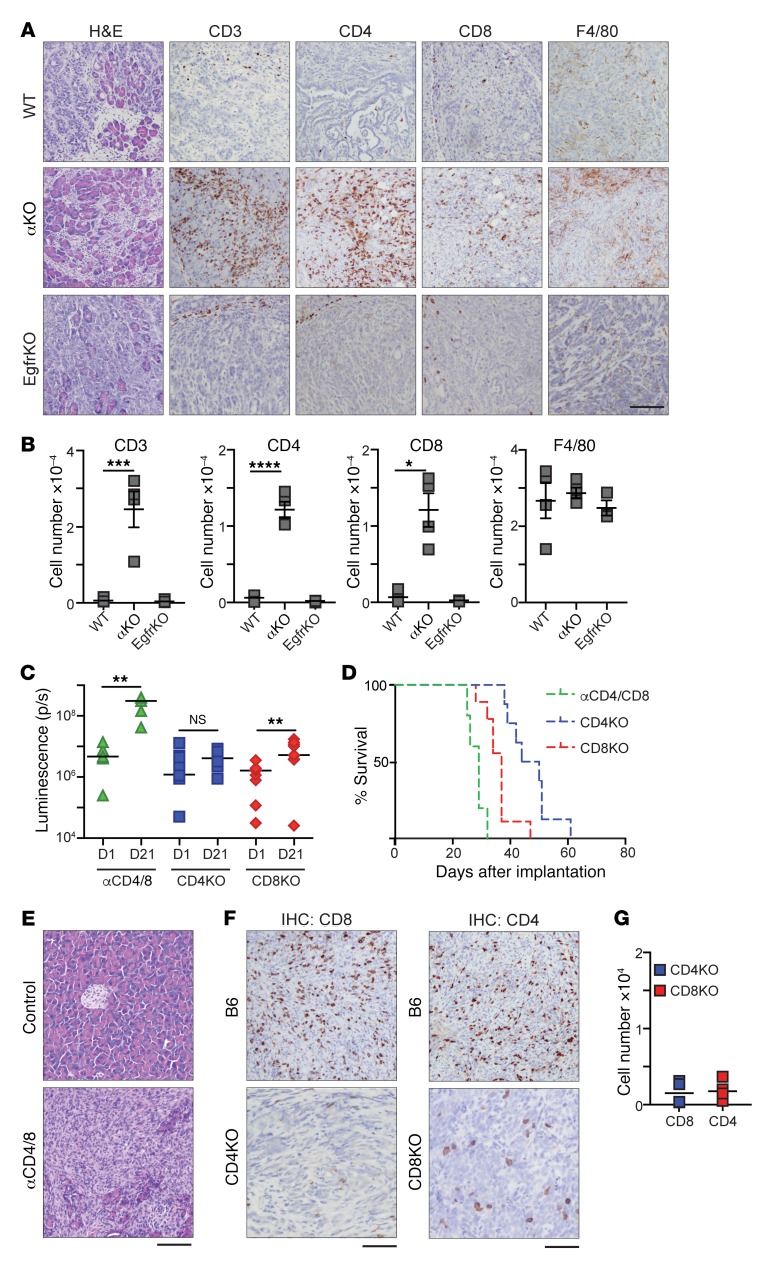
T cell infiltration of αKO tumors and the requirement of CD4^+^ and CD8^+^ T cells for αKO tumor regression. (**A** and **B**) WT, αKO, or EgfrKO cells (0.5 million) were implanted in the head of the pancreas of B6 mice, and pancreata were harvested 10 days later (EgfrKO, *n* = 3; WT and αKO, *n* = 4). (**A**) Sections were stained with H&E, or IHC was performed with the indicated antibodies. Representative sections are shown. Scale bar: 100 μm. (**B**) Quantification of tumor-infiltrating cells positive for CD3, CD4, CD8, or F4/80. Bars indicate mean ± SEM. WT versus αKO, ***P = 0.0008, ****P = 0.0001, and *P = 0.0005 for CD3, CD4, and CD8, respectively (1-way ANOVA with Bonferroni’s post hoc test). (**C**–**G**) αKO cells (0.5 million) were implanted in the head of the pancreas of B6 mice injected with neutralizing CD4 and CD8 antibodies (αCD4/8, green) or PBS (control), or CD4KO (blue squares) or CD8KO (red diamonds) mice. (**C**) Quantification of luciferase signals from IVIS images of each mouse. Bars indicate median. **P < 0.005 (2-tailed Wilcoxon signed-rank test). (**D**) Kaplan-Meier survival curves for αCD4/8 (*n* = 5; median survival: 29 days), CD4KO (*n* = 9; median survival: 47 days), and CD8KO (*n* = 8; median survival: 37 days) mice implanted with αKO cells. *P* = 0.0017 (log-rank test, CD4KO vs CD8KO). (**E**) H&E-stained pancreatic sections from αCD4/8 and control mice. (**F**) IHC staining of pancreatic sections with antibodies against CD4 or CD8. Pancreata were collected from CD4KO or CD8KO mice at the humane endpoint. For comparison, sections of pancreata collected 10 days after implantation of B6 mice with 0.5 million αKO cells are shown. Scale bars: 100 μm. (**G**) Quantification of tumor-infiltrating CD4^+^ or CD8^+^ T cells from pancreatic sections.

To investigate if a host immune response might be responsible for the regression of αKO tumors in our mouse model, we implanted WT, αKO, or EgfrKO tumor cells in the pancreas of B6 mice and sacrificed the animals 10 days later. H&E staining of pancreatic sections from all 3 groups showed substantial areas of normal pancreas interspersed with tumors (Figure 2A). Immunohistochemistry showed that αKO tumors were infiltrated with large numbers of CD3^+^, CD4^+^, and CD8^+^ T cells (Figure 2, A and B). Similar to most human pancreatic tumors, WT and EgfrKO tumors were associated with peritumoral T cells, but few T cells were seen infiltrating the tumors (Figure 2, A and B). We did not observe a significant difference in the number of F4/80^+^ macrophages infiltrating the tumors in the 3 groups (Figure 2, A and B). We also harvested pancreata from B6 mice 10 days after implantation with αKD DT10022 cells, and immunohistochemistry showed that these tumors were also infiltrated with CD3^+^ and CD8^+^ T cells (Supplemental Figure 3D).

To test if tumor-infiltrating T cells are responsible for tumor regression, we implanted αKO KPC cells in the pancreas of B6 mice whose T cells were depleted by injection of neutralizing antibodies to CD4 and CD8. Depletion of circulating T cells was confirmed by flow cytometry (Supplemental Figure 4A). Unlike the results using immunocompetent B6 mice, IVIS imaging showed that αKO tumors grew in these T cell–deficient animals (Supplemental Figure 4B). The increase in median tumor volume from day 1 to day 21 was 65.7-fold (Figure 2C). All mice that received neutralizing antibodies died due to tumor growth, with a median survival of 29 days (Figure 2, D and E, and Table 1). To determine the specific subtype of T cells involved in tumor regression, we implanted αKO cells in the pancreas of mice genetically engineered to lack CD4^+^ or CD8^+^ T cells (referred to as CD4KO or CD8KO mice, respectively). αKO tumors did not regress in CD4KO or CD8KO mice and all of the animals became moribund (Figure 2, C and D, Supplemental Figure 4B, and Table 1). Tumor growth was slower in the CD4KO mice, and they survived longer than the CD8KO animals (median survival of 47 days vs 37 days, respectively). Immunohistochemistry showed a decrease in the number of tumor-infiltrating CD8^+^ T cells in CD4KO mice and fewer tumor-infiltrating CD4^+^ T cells in tumors in CD8KO mice as compared with tumors in B6 animals (Figure 2, F and G). These results suggest that both CD4^+^ and CD8^+^ T cells are essential and that they cooperate with each other to infiltrate αKO tumors and cause tumor regression.

### Adoptive T cell transfer protects SCID mice from αKO KPC tumors.

**Figure 3:**
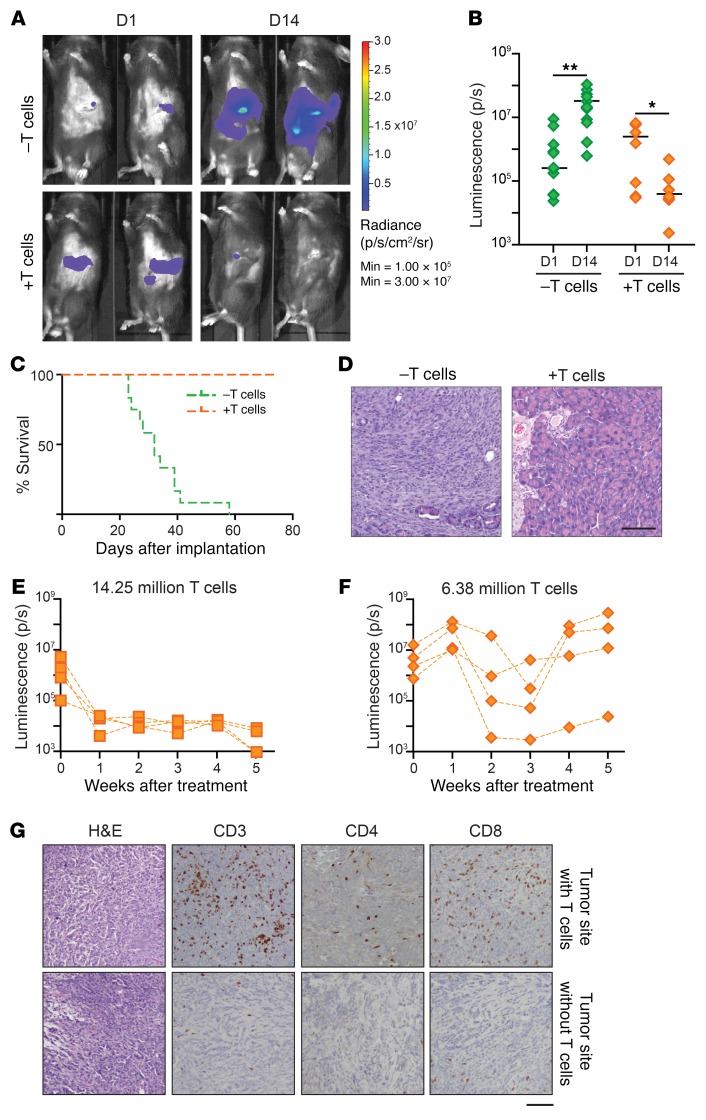
Adoptive T cell transfer protects SCID mice from implanted αKO tumors. (**A**–**D**) SCID mice received either no pretreatment (–T cells, *n* = 12) (green) or adoptive transfer of 5 million T cells harvested from B6 mice previously implanted with αKO cells (+T cells, *n* = 8) (orange). One day later, αKO cells (0.5 million) were implanted in the head of the pancreas of each animal. (**A**) Tumor growth was monitored by IVIS imaging. Representative images of 2 mice in each group are shown. (**B**) Quantification of luciferase signals from each mouse. Bars indicate median. *P = 0.0156 and **P = 0.0024 (Wilcoxon signed-rank test). (**C**) Kaplan-Meier survival curves. Median survival: –T cells, 32 days; +T cells, all were alive at day 80. *P* < 0.00001 (log-rank test). (**D**) Representative H&E-stained pancreatic sections. Pancreata were collected at the humane endpoint (–T cells) or 4 months after tumor implantation (+T cells). Scale bar: 100 μm. (**E**–**G**) 0.5 million αKO KPC cells were implanted in the head of the pancreas of SCID mice (*n* = 8). Four days later (*t* = 0), the mice were imaged by IVIS and then treated with 14.25 million or 6.38 million T cells from a convalescent B6 mouse previously implanted with αKO cells (*n* = 4 per group). Tumor growth was monitored by IVIS imaging. (**E** and **F**) Quantification of luciferase signals over time. (**G**) Mice treated with 6.38 million T cells were sacrificed at week 5 and their pancreata were collected for histology. Representative H&E and IHC images of different tumor sites from the same mouse are shown. Scale bar: 100 μm.

We next investigated if T cells from a convalescent B6 mouse (implanted with αKO cells and recovered for 6 months; see Figure 1G) can target and kill αKO cells implanted in mice with SCID that lack functional T and B cells. We first implanted αKO cells in the head of the pancreas of a control group of SCID mice. The tumors grew rapidly and killed all of the animals (Figure 3, A–C, and Table 1). A second group of SCID mice was implanted with αKO cells 1 day after receiving an adoptive transfer of CD90.2^+^ T cells enriched from the spleens of convalescent mice. The αKO tumors regressed in these animals (Figure 3, A and B, and Supplemental Figure 5A) and all of the mice survived for more than 80 days without overt signs of illness (Figure 3C and Table 1). H&E-stained pancreatic sections showed that the control group of SCID mice had large tumors with little normal tissue, whereas mice that received T cells had completely normal pancreas 4 months after implantation (Figure 3D).

We next asked if adoptive T cell transfer is an effective treatment for established αKO tumors. Two groups of SCID mice were implanted with αKO cells in the pancreas. After determining tumor size 4 days later by IVIS imaging (Figure 3, E and F; *t* = 0), 1 group of animals was given 14.25 million T cells from convalescent mice and the second group was treated with 6.38 million T cells. Mice that received 14.25 million T cells showed complete tumor regression after 1 week (Figure 3E and Supplemental Figure 5B) and all of the animals survived for more than 10 weeks. In mice that received fewer T cells, the tumors regressed but started growing again after 3 to 4 weeks (Figure 3F and Supplemental Figure 5B). We collected pancreata from the animals treated for 5 weeks with 6.38 million T cells and examined pancreatic sections by immunohistochemistry. Some of the αKO tumors were infiltrated by CD4^+^ and CD8^+^ T cells (Figure 3G, top panels), whereas others from the same mouse were scarcely infiltrated with T cells (Figure 3G, bottom panels). These results suggest that T cells from mice previously exposed to the αKO tumor can be used to induce tumor regression in another animal as long as the number of T cells relative to tumor cells is adequate. Indeed, the significance of T cell number and the time of adoptive T cell transfer to eradicate tumors has been reported (27).

### Pik3ca-dependent suppression of MHC I and CD80 levels contributes to immune evasion of WT KPC tumors.

**Figure 4:**
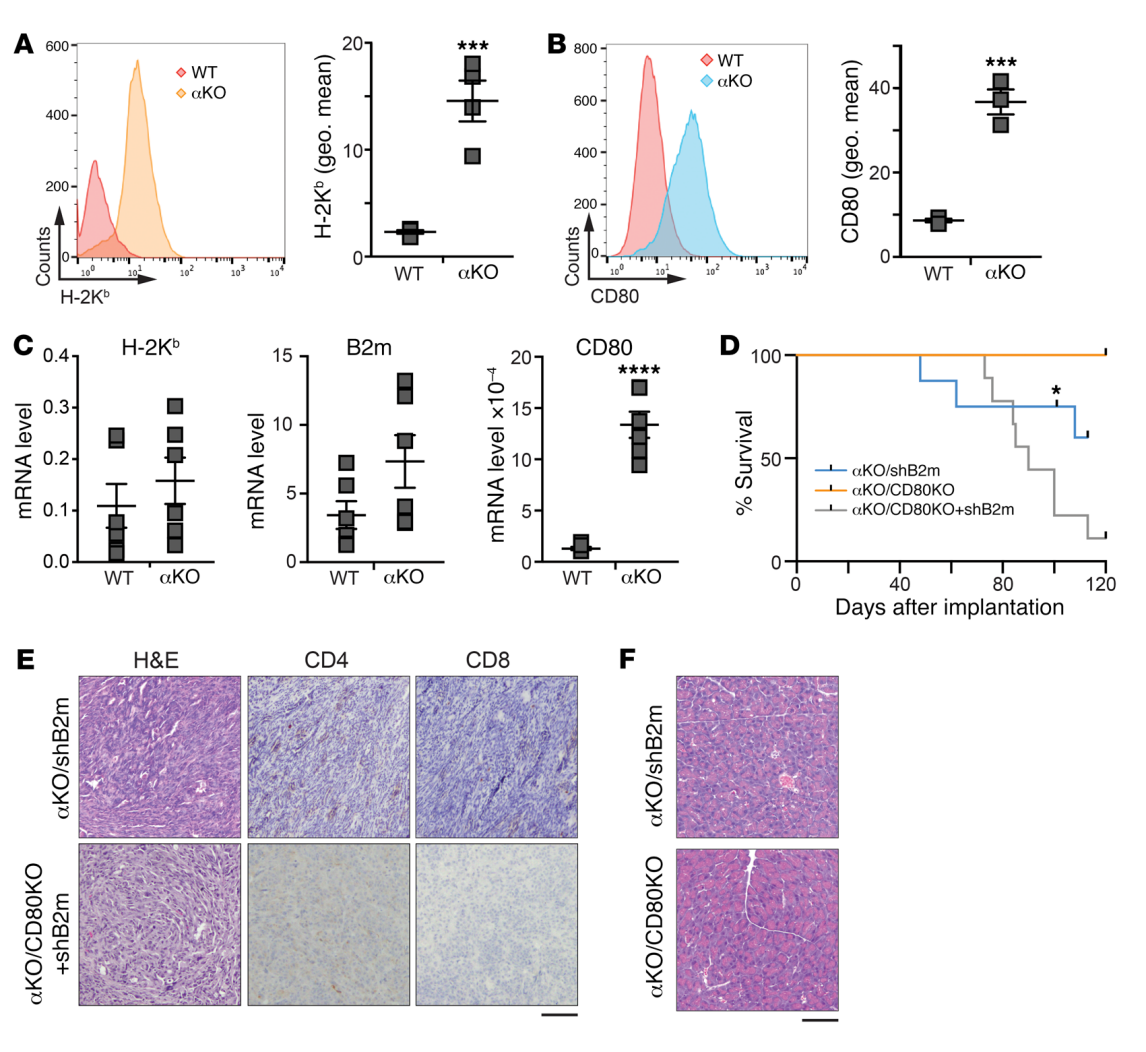
PIK3CA regulates cell surface expression of MHC I and CD80 in KPC cells. Flow cytometric analysis of cell surface levels of (**A**) H-2K^b^ and (**B**) CD80 in WT and αKO cells. The left panels show representative histograms and the right graphs show the mean ± SEM of the geometric means (geo. mean) of each flow cytometry distribution. H-2K^b^, *n* = 4; CD80, *n* = 3. ***P = 0.0007 (unpaired *t* test). (**C**) qRT-PCR analysis of mRNA expression of H-2K^b^, B2m, and CD80 in WT and αKO cells. Gene expression changes were normalized to *Hprt*. Graph shows mean ± SEM (*n* = 6). ****P < 0.0001 (unpaired *t* test). (**D**–**F**) B6 mice were implanted with 0.5 million αKO/shB2m, αKO/CD80KO, or αKO/CD80KO+shB2m cells in the head of the pancreas and tumor growth was monitored by IVIS imaging. (**D**) Kaplan-Meier survival curves for αKO/shB2m, αKO/CD80KO, and αKO/CD80KO+shB2m mice. *A single mouse was euthanized for histology and removed from the study. Median survival: αKO/shB2m, 110.5 days; αKO/CD80KO+shB2m, 87.5 days. All αKO/CD80KO mice were alive at 115 days. *P* = 0.0026 (log-rank test). (**E**) Representative H&E and IHC images of pancreatic sections from αKO/shB2m or αKO/CD80KO+shB2m mice that died of tumor progression. Scale bar: 100 μm. (**F**) Representative H&E-stained pancreatic sections from a single αKO/shB2m mouse or αKO/CD80KO mice euthanized at 101 or 160 days, respectively. Scale bar: 100 μm.

Priming of CD4^+^ helper T cells requires binding of the TCR to antigens presented on MHC class II (MHC II), which is normally expressed in immune cells, but it has been detected on some cancer cells (28, 29). We wondered if a difference in expression of MHC I, MHC II, or CD80 might explain the different host immune responses to implanted αKO or WT KPC cells. Indeed, flow cytometry showed that cell surface expression of the MHC I heavy chain (H-2K^b^ in B6 mice) was 6.4 times higher in αKO KPC cells than in WT cells (Figure 4A; average geometric means of 14.7 vs 2.3, respectively). We also found that cell surface CD80 was 4.4 times higher in αKO cells than in WT cells (Figure 4B; average geometric mean, 36.8 vs 8.4, respectively). H-2K^b^ and B2m mRNA levels tended to be higher in αKO cells, but the differences were not statistically significant. By contrast, CD80 mRNA levels were significantly higher in αKO cells (Figure 4C). Cell surface expression of MHC II was not detected in either cell line (Supplemental Figure 6A). A significant increase in CD80 surface expression was also seen in αKD cells as compared with WT DT10022 cells (Supplemental Figure 3E). These results suggest that *Pik3ca*-dependent suppression of MHC I and CD80 in WT KPC cells may allow the tumors to evade lysis by CD8^+^ T cells. CD4^+^ T cells may provide helper functions to the CD8^+^ T cells that infiltrate αKO tumors.

The roles of MHC I and CD80 in immune-mediated regression of αKO tumors were furthered investigated using αKO cell lines with reduced expression of these proteins. CRISPR/Cas9 was used to target *Cd80* in αKO cells to generate αKO/CD80KO cells. MHC I cannot be knocked out completely in cells used for implantation experiments because natural killer cells recognize and kill cells that lack the self MHC I. Therefore, we used shRNA to knock down the level of B2m in αKO or αKO/CD80KO cells to produce αKO/shB2m or αKO/CD80KO+shB2m cell lines, respectively. Flow cytometry confirmed a reduction in CD80 and/or H-2K^b^ on the cell surface in the appropriate cell lines (Supplemental Figure 6B). We observed no significant difference in proliferation rate of the 3 cell lines in 2D culture as compared with αKO cells (Supplemental Figure 6C). Implantation of the 3 cell lines in the pancreas of B6 mice resulted in initial tumor growth in almost all of the animals over the first week (Supplemental Figure 7). Three of 8 mice implanted with αKO/shB2m cells died of tumor progression, and necropsy showed a solid mass in the pancreas and metastases to the peritoneum, diaphragm, liver, and lungs. The remaining 5 mice in this group lived for more than 100 days (Figure 4D and Table 1). All of the animals implanted with αKO/CD80KO cells survived more than 100 days, whereas all of the mice implanted with αKO/CD80KO+shB2m cells eventually died from tumor progression (Figure 4D and Table 1). Examination of pancreatic tissue from αKO/shB2m or αKO/CD80KO+shB2m mice that succumbed to tumor progression showed large tumors that were devoid of infiltrating CD4^+^ or CD8^+^ T cells (Figure 4E). No tumors were detected in pancreatic tissue from 1 αKO/shB2m mouse or αKO/CD80KO mice that were sacrificed at 101 days or 160 days, respectively (Figure 4F). These results indicate that upregulation of both MHC I and CD80 contributes to regression of αKO KPC tumors. The extended survival of mice with αKO/CD80KO+shB2m tumors as opposed to WT KPC tumors suggests that other tumor-specific or host-related factors also play a role in tumor progression.

### AKT acting downstream of PIK3CA is responsible for immune evasion of KPC cells.

**Figure 5:**
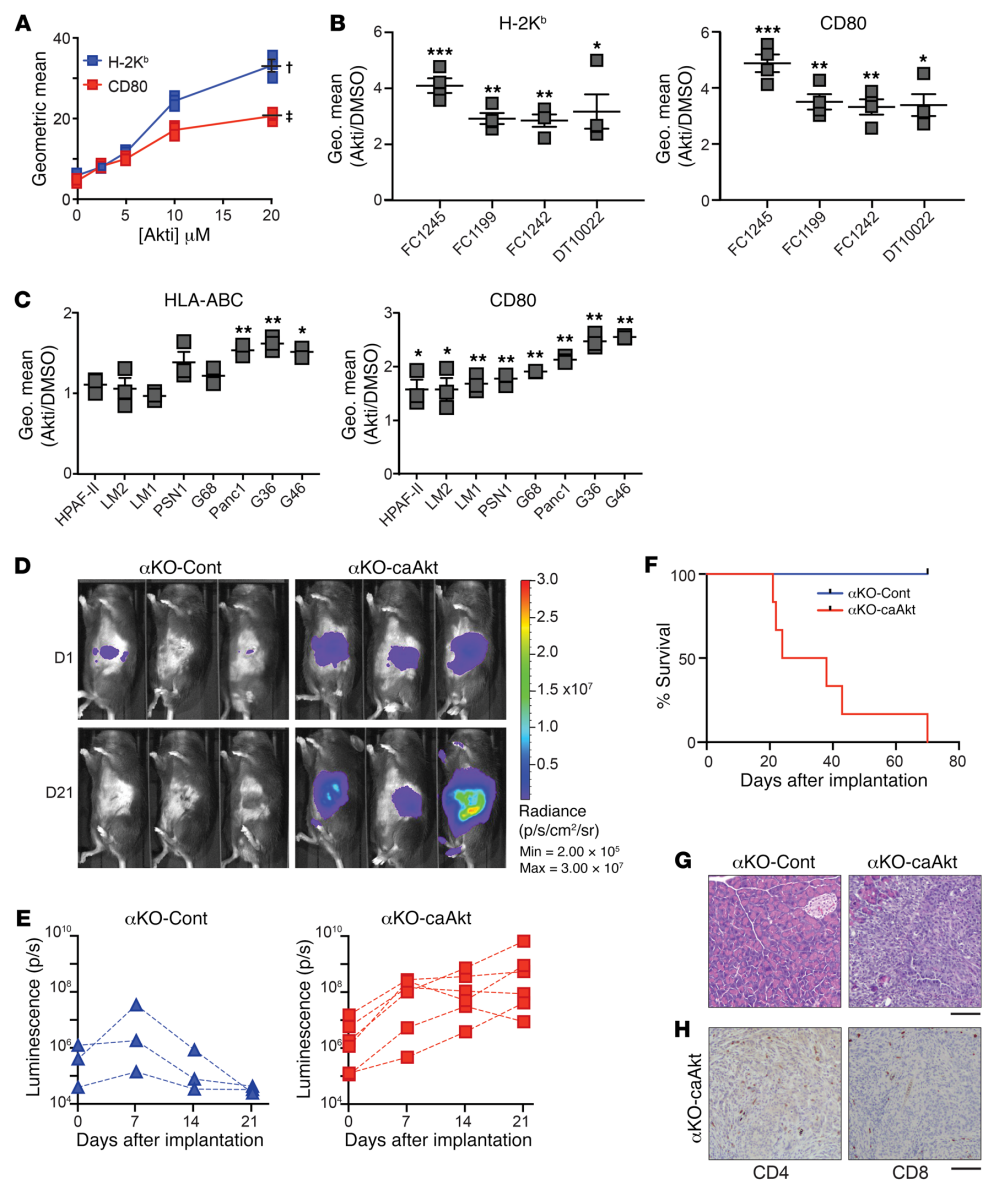
PIK3CA regulation of CD80 and MHC I is mediated by AKT signaling. (**A**) WT KPC cells were treated with increasing concentrations of AKT inhibitor (Akti) for 48 hours. Cell surface levels of CD80 and H-2K^b^ were quantified by flow cytometry. The mean ± SEM of the geometric means of each flow cytometry distribution is plotted versus inhibitor concentration (*n* = 3 for each inhibitor concentration). ^†^*P* = 0.0031 (H-2K^b^) and ^‡^*P* = 0.0018 (CD80) for DMSO versus 20 μM Akti (2-tailed paired *t* test). (**B**) Four parental KPC cell lines were treated with 10 μM Akti for 48 hours. Cell surface levels of H-2K^b^ (left) and CD80 (right) were determined by flow cytometry (*n* = 4). The graphs show fold change (mean ± SEM) over DMSO. *P < 0.05, **P < 0.005, ***P < 0.0005. (**C**) Human pancreatic cell lines were treated with 10 μM Akti for 48 hours. Cell surface levels of HLA-ABC (left) and CD80 (right) were determined by flow cytometry (*n* = 3). The graph shows fold change (mean ± SEM) over DMSO. *P < 0.05, **P < 0.005. (**D**–**H**) αKO-Cont or αKO-caAkt cells (0.5 million) were implanted in the head of the pancreas of B6 mice. (**D**) Representative IVIS images showing tumor size on days 1 and 21. (**E**) Quantification of luciferase signals in each mouse. αKO-Cont, *n* = 3 and αKO-caAkt, *n* = 6. (**F**) Kaplan-Meier survival curves. Median survival: αKO-caAkt, 31 days. All αKO-Cont mice were alive at 71 days. *P* = 0.011 (log-rank test). (**G**) Representative H&E-stained pancreatic sections from mice implanted with αKO-Cont cells (euthanized at 71 days) or αKO-caAkt cells (postmortem). (**H**) Representative IHC images of pancreatic sections from mice implanted with αKO-caAkt cells (postmortem). For **G** and **H**, *n* = 3. Scale bar: 100 μm.

Because AKT activity is significantly higher in WT KPC cells than in αKO cells (Supplemental Figure 1), we wondered if AKT acts downstream of PIK3CA to suppress MHC I and CD80 levels. Flow cytometry of WT cells treated with increasing concentrations of an AKT inhibitor (Akti) showed a dose-dependent increase in expression of H-2K^b^ and CD80 (Figure 5A). The increases in average geometric mean at 20 μM Akti versus DMSO were 5.5-fold (H-2K^b^) and 4.4-fold (CD80). These increases in expression are comparable to the different expression levels in αKO versus WT KPC cells (6.3-fold [H-2K^b^] and 4.4-fold [CD80]) (Figure 4, A and B). Parental FC1245 cells and 3 additional KPC cell lines (FC1199, FC1242, and DT10022) all showed an increase in both H-2K^b^ and CD80 after treatment with 10 μM Akti (Figure 5B). The difference in average geometric mean at 10 μM Akti versus DMSO was highest in FC1245 cells (4-fold for H-2K^b^ and 4.8-fold for CD80). In the other cell lines, the differences in average geometric mean for H-2K^b^ and CD80 were statistically significant and varied from 2.8- to 3-fold for H-2K^b^ and 3.3- to 3.5-fold for CD80 (Figure 5B).

Akti treatment also significantly increased the cell surface expression of MHC I (HLA-ABC in human) in 3 of 8 human PDAC cell lines (Figure 5C). This result is not surprising, since there are potentially many mechanisms by which MHC I can be downregulated. For example, cancer cells with biallelic loss of MHC I will not respond to AKT inhibition. Somewhat surprisingly, all of the human PDAC cell lines tested showed upregulated CD80 expression after Akti treatment (Figure 5C). Western blotting of WT KPC and human PDAC cell lines showed that Akti treatment reduced the level of phospho-AKT (Supplemental Figure 8).

We next tested if increasing AKT activity in αKO pancreatic tumors allows them to evade immune surveillance. Western blotting showed that αKO cell lines stably expressing a constitutively active form of AKT (αKO-caAkt cells) have high levels of phospho-AKT as compared with vector control cells stably expressing the control vector (αKO-Cont cells; Supplemental Figure 9A). αKO-caAkt cells proliferated about 1.4 times faster than αKO-Cont cells in 2D culture (Supplemental Figure 9B). When implanted in the pancreas of B6 mice, the αKO-Cont tumors grew over the first 7 days and then regressed, and all of the mice lived for more than 70 days (Figure 5, D–F, and Table 1). In contrast, the αKO-caAkt cells grew rapidly, formed large tumors, and killed all of the animals (Figure 5, D–F, and Table 1). Mice that died from implanted αKO-caAkt cells had large pancreatic tumors and extensive metastases to the liver, lungs, and colon. Some mice also had metastases to the diaphragm and peritoneum. Pancreatic sections showed tumors devoid of intratumoral CD4^+^ or CD8^+^ T cells, although T cells were present in tissue surrounding the tumors (Figure 5, G and H). These results are similar to what we observed with WT KPC cells implanted in B6 mice (Figure 1, D–F). Mice implanted with αKO-Cont cells were sacrificed 71 days after implantation. H&E-stained pancreatic sections showed no abnormalities (Figure 5G). These results suggest that PIK3CA-AKT signaling mediates immune evasion in WT KPC cells.

## Discussion

Pancreatic tumors in patients are well known for their ability to exclude T cells (30–32), and limited T cell infiltration is also a prominent feature of pancreatic tumors in *Kras^LSL–G12D/+^ Trp53^LSL–R172H/+^ Pdx1-Cre* mice (30, 33, 34). The presence of tumor-infiltrating cytotoxic T cells is correlated with increased survival of patients with pancreatic cancer (3–6). The salient finding of this study is that PIK3CA-AKT signaling regulates the susceptibility of KPC pancreatic tumors to T cell antitumor responses that result in infiltration and elimination of the tumor. *Pik3ca*-null tumors contained significantly more tumor-infiltrating T cells than WT tumors, and the αKO tumors regressed only in mice with functional T cells.

MHC I is part of the machinery that presents processed antigenic peptides to cytotoxic CD8^+^ T cells. Suppression of cell surface MHC I is a well-documented mechanism used by some tumors to evade the immune system (8). Conversely, high MHC I expression in triple negative breast tumors is correlated with increased tumor-infiltrating lymphocytes and better prognosis in patients (35). One important phenotype caused by *Pik3ca* loss in KPC cells is upregulation of MHC I on the cell surface. Treatment of WT KPC cells or some human PDAC cell lines with an AKT inhibitor caused a similar change. To our knowledge, this is the first report that PIK3CA-AKT signaling negatively regulates MHC I in cancer cells. Earlier studies identified some other pathways that regulate MHC I. A human kinome shRNA interference-based screen was used to discover several kinases that positively or negatively regulate MHC I expression (36). MAP2K1 (also called MEK1) and EGFR were validated as negative regulators by showing that treatment of several cancer cell lines with inhibitors of either enzyme increased mRNA levels and cell surface expression of HLA-A and B2m (36). Cytokines such as IFN-γ secreted by T helper cells have been shown to upregulate MHC I expression via JAK/STAT signaling (37). Interestingly, a key factor mediating HIV immune escape is the HIV-1 protein Nef, which acts through PI3K to downregulate trafficking of MHC I to the cell surface (38).

We also analyzed The Cancer Genome Atlas (TCGA) database to assess the relationship between PI3K pathway status and the expression levels of CD80 and B2M genes in patients with PDAC. The analysis showed that PTEN expression was positively correlated with CD80 and B2M gene expression, whereas phospho-AKT protein levels were inversely correlated with the expression of CD80 and B2M genes (Supplemental Figure 10). Further studies are needed to identify the mechanisms downstream of PIK3CA and AKT that are responsible for downregulating MHC I and CD80 in pancreatic cancer cells.

A second important phenotype caused by *Pik3ca* loss or AKT inhibition in pancreatic cancer cells is upregulation of CD80 on the cell surface. CD80 binds to CD28 on T cells and provides a costimulatory signal that is indispensable for T cell activation. Most tumors do not express CD80, but engineered expression of endogenous or exogenous CD80 on tumor cells has been shown to enhance antitumor T cell responses and promote tumor regression (12, 39, 40). Cytotoxic T lymphocyte antigen 4 (CTLA-4) is another CD80 receptor expressed on T cells. CD80 ligation with CTLA-4, unlike CD28, initiates a suppressive signaling cascade that leads to T cell exhaustion (41). Upregulating CD80 on tumor cells potentially can result in T cell exhaustion and augment tumor progression. However, our results show that αKO KPC tumors were efficiently cleared by the immune system, suggesting that inhibitory signals through CTLA-4 are minor. We observed elevated levels of PD-L1 and PD-L2 in αKO cells and in Akti-treated WT cells (Supplemental Figure 11). PD-L1 and PD-L2 expressed on tumor cells potentially can suppress T cell antitumor responses (42), but they do not exert a dominant inhibitory effect in our model. Interestingly, an increase in PD-L1 and PD-L2 in gastric cancers was correlated with increased T cell infiltration of the tumors and better prognosis (43). Deleting CD80 was not sufficient to convert “hot” αKO KPC tumors with infiltrating T cells into “cold” tumors that exclude T cells. Knock down of MHC I was more successful in this regard, but αKO/shB2m tumors progressed in only 3 of 8 implanted animals. The combined actions of MHC I downregulation plus CD80 silencing were required to reverse the highly immunogenic phenotype of αKO KPC tumors. Because the median survival of mice with αKO/CD80KO+shB2m tumors (87.5 days) was much longer than that of mice with WT KPC tumors (16 days), we suspect that additional *Pik3ca*-regulated immune modulators, perhaps including cytokines, contribute to tumor immunogenicity.

Our data using CD4KO and CD8KO mice indicate that both CD4^+^ and CD8^+^ T cells are essential for αKO KPC tumor regression. Without one subtype of T cell, the other subtype was less able to infiltrate the tumors. CD4^+^ T helper (Th) cells secrete cytokines and chemokines such as IFN-γ and IL-2 that provide helper functions to B cells, APCs, and the cytotoxic CD8^+^ T cells that ultimately lyse tumors (27, 44). Especially in MHC II–negative tumors, CD4^+^ T cells are critical during the effector phase of the antitumor response. Mice depleted of CD4^+^ T cells prior to tumor challenge were unable to clear the tumor (45). In the context of PDAC, IFN-γ–secreting Th1 cells were shown to slow tumor growth by stimulating the proliferation of CD8^+^ cytotoxic T cells, while IL-5–secreting Th2 cells promoted tumor growth by inhibiting CD8^+^ cells (46). CD4^+^ T cells may also acquire cytotoxic activity in vivo as seen in some melanomas (47, 48), and can play a role in generating and maintaining memory CD8^+^ T cells (49, 50). Adoptive T cell transfers of both CD4^+^ and CD8^+^ T cells have been shown to be more effective than either subtype alone (27, 51). CD4^+^ and CD8^+^ T cell infiltrate in PDAC patient tumors is primarily of the effector memory type with few naive T cells (32). Because CD4^+^ T cells can alter the tumor microenvironment and facilitate CD8^+^ T cell trafficking and function at the tumor site (27, 52), our finding that CD4^+^ T cells are vital for the recruitment of CD8^+^ T cells to the tumor site is perhaps not unexpected. Surprisingly, we observed fewer tumor-infiltrating CD4^+^ T cells in mice lacking CD8^+^ T cells. It is unclear how CD8^+^ T cells might affect the trafficking of CD4^+^ T cells to the tumor site. Additional studies are necessary to address the trafficking and function of CD4^+^ T cells in the immune response to αKO KPC tumors.

Although PIK3CG is expressed mainly in hematopoietic cells, it was reported to be present at high levels in human pancreatic cancer tissue. Downregulation of PIK3CG slightly slowed the proliferation of PDAC cell lines in culture (53). A subsequent study showed that genetic ablation or systemic pharmacological inhibition of PIK3CG led to activation of T cells and slower PDAC growth in vivo (21). These antitumor effects were attributed to suppression of PIK3CG in macrophages leading to suppression of cytotoxic T cells and not due to decreased PIK3CG in tumor cells. Genetic or pharmacologic downregulation of PIK3CG altered the transcriptional program of tumor-infiltrating macrophages to promote cytotoxic T cell infiltration of pancreatic tumors, thus extending survival of the mice (21, 54, 55). However, unlike ablation of *Pik3ca* in KPC tumor cells, all of the animals with downregulated PIK3CG eventually died from pancreatic cancer progression (21). PIK3CD is expressed mainly in leukocytes and is critical for the differentiation and function of effector T cells and CD4^+^ Tregs (56). Suppression of PIK3CD in Tregs protected host mice from a broad range of transplanted cancers (20), but it is unclear if downregulation of PIK3CD slowed pancreatic cancer growth in vivo (57). Several immunotherapeutic strategies are being investigated to promote infiltration of T cells into pancreatic tumors (10). Our results indicate that PIK3CA may be a potential drug target to increase T cell immunogenicity of pancreatic cancer. Treatment with a pan-isoform PI3K inhibitor did not reduce the tumor volume of KPC cells or patient-derived xenografts implanted in mice (58). Based on our current understanding, systemic pharmacological inhibition of all PI3K isoforms should enhance the immunogenicity of the pancreatic tumors by upregulating MHC I and CD80, but inhibitory effects on cytotoxic T cells will negate these beneficial changes.

In conclusion, we have shown that PIK3CA-AKT signaling in WT KPC tumors reduces the cell surface expression of MHC I and CD80 to promote immune evasion. Genetic ablation of *Pik3ca* counteracts these effects to render pancreatic tumors more immunogenic and thus more susceptible to T cell clearance. Some tumor cells have been demonstrated to directly present tumor antigens to CD8^+^ T cells (59, 60). Whether αKO cells directly present tumor antigens on their surface is still unclear. Based on our results, we believe that abrogating PIK3CA-AKT signaling in tumor cells attracts both CD4^+^ and CD8^+^ T cells to infiltrate the tumors. CD4^+^ and CD8^+^ T cells act synergistically to cause tumor regression. WT KPC tumors are surrounded by T cells and evade the immune system due to PIK3CA-mediated downregulation of MHC I and CD80. αKO tumors, on the other hand, are infiltrated with T cells that can recognize and lyse the tumors. Our results could pave the way for a robustly effective treatment for pancreatic cancer, which has so far been lacking.

## Methods

### Cell lines.

FC1245, FC1242, FC1199, and DT10022 cells (25) were a gift from David Tuveson (Cold Spring Harbor Laboratory, Cold Spring Harbor, New York). Unless otherwise noted, FC1245 cells were used for all of the studies. The cells were infected with lentiviral particles containing firefly luciferase under the control of a CMV promoter (Cellomics Technology, PLV-10064). Cells were selected 48 hours after infection with 1.5 mg/mL G418 and screened for luciferase expression on the IVIS Lumina III imaging system (Xenogen) to generate stable luciferase-expressing cells referred to as WT KPC cells. SNP analysis of WT KPC showed that the cells have a 99.63% C57BL/6J and 0.37% C57BL/6NJ genetic background with a single T/A polymorphism on chromosome 8. DNA sequencing of exon 1 of the *Kras* gene confirmed that the cells have a G-to-D mutation at codon 12. We did not detect a WT copy of *Kras* (Supplemental Figure 12). Loss of the WT allele is common in cell lines derived from genetically engineered mouse models and patients with PDAC or other cancers (61–64). Loss of WT *KRAS* is frequently associated with amplification of the mutant allele and an increase in aggressiveness and migration of PDAC and other cancers (61–65). To generate *Pik3ca*^–/–^ (αKO) KPC cell lines, WT KPC cells were transfected concurrently with *Pik3ca* CRISPR/Cas9 KO and HDR plasmids (Santa Cruz Biotechnology, sc-422231 and sc-422231-HDR). *Pik3ca* knockdown (αKD) DT10022 cells were made using the same plasmids. To generate the *Egfr*^–/–^ (EgfrKO) cell line, WT KPC cells were transfected concurrently with *Egfr* CRISPR/Cas9 KO and HDR plasmids (Santa Cruz Biotechnology, sc-420131 and sc-420131-HDR). Transfected cells were selected with 5 μg/mL puromycin and RFP^+^ cells were collected using fluorescence-activated cell sorting (FACS) on a FACSAria (BD Biosciences). RFP^+^ cells were serially diluted in a 96-well plate to generate single cell clones. Clones that showed an absence of PIK3CA or EGFR protein on Western blots were retained. αKO KPC cells lacking CD80 (referred to as αKO/CD80KO) were generated by transfecting αKO cells with B7-1 CRISPR/Cas9 KO plasmid (Santa Cruz Biotechnology, sc-419570). Cells were collected 48 hours after transfection using StemPro Accutase (Thermo Fisher Scientific), incubated with FcR block, and stained for CD80. CD80-null cells were collected by FACS. Single cell clones were generated by serial dilution and confirmed by flow cytometry. αKO cells with reduced levels of B2m (referred to as αKO/shB2m) were generated by infecting αKO cells with B2m shRNA lentiviral particles (Santa Cruz Biotechnology, sc-29593-V). Cells were stained for H-2K^b^ 48 hours after infection, and cells with low levels of H-2K^b^ were collected by FACS. αKO/CD80KO cells were infected with β2-microglobulin shRNA lentiviruses and FACS sorted to generate αKO KPC cells without CD80 and with low levels of H-2K^b^ (referred to as αKO/CD80KO+shB2m). αKO-caAkt and αKO-Cont cells were generated by infecting αKO cells with lentiviral particles expressing a constitutively active human Akt1 mutant and EGFP or EGFP alone, respectively (pHRIG-Akt1; Addgene plasmid 53583; gift from Heng Zhao) (66). Cells expressing EGFP were collected 48 hours after infection using FACS, and mutant Akt1 expression was confirmed by Western blotting. All murine cell lines were cultured in DMEM media containing 10% fetal bovine serum and 1% penicillin/streptomycin at 37°C and 5% CO_2_. Human pancreatic cancer cell lines Panc1, HPAF-II, and PSN1 were purchased from ATCC and cultured using recommended conditions. Patient-derived human pancreatic cancer cell lines were previously described and cultured as recommended (67).

### 2D cell proliferation assay.

Cells (5,000) were plated in triplicate in a 6-well plate. On days 1 through 4, cells were trypsinized, mixed with an equal volume of trypan blue, and counted using the Countess II automated cell counter (Life Technologies). Cell number was plotted against time using GraphPad Prism 7.

### 3D cell culture.

3D cultures used previously described protocols (68). Briefly, 1,000 cells in DMEM containing 10% FBS were mixed with methylcellulose stock solution to a final concentration of 0.24% methylcellulose. Cells were cultured by the hanging drop method on the lid of a 96-well plate. To prevent evaporation, PBS was added to the plate. The plates were incubated at 37**°**C in a 5% CO_2_ atmosphere. The cells were imaged on day 5 using brightfield microscopy with a ×40 objective.

### Immunoblotting.

Cell lysates were prepared in RIPA buffer (50 mM HEPES, pH 7.4, 10 mM sodium pyrophosphate, 50 mM NaCl, 50 mM NaF, 5 mM EDTA, 1 mM sodium orthovanadate, 0.25% sodium deoxycholate, 1% NP40, 1 mM PMSF, and protease inhibitor cocktail; MilliporeSigma P8340) and cleared by centrifugation at 4**°**C. Cell proteins were separated by SDS-PAGE and transferred to nitrocellulose membranes by semi-dry transfer, and the membranes were incubated with primary antibodies in Tris-buffered saline plus 0.1% Tween 20. After incubation in HRP-linked secondary antibody followed by ECL reagent Western Lightning Plus-ECL (PerkinElmer) or SuperSignal West Femto (Thermo Fisher Scientific), signals were detected using a FluorChem E imager (ProteinSimple). To detect multiple proteins on the same membrane, HRP was inactivated by incubating membranes in 30% H_2_O_2_ for 30 minutes (69), or the membranes were stripped for 30 minutes at 50°C in 62.5 mM Tris, pH 6.7, 2% SDS, and 100 mM 2-mercaptoethanol prior to reprobing with another antibody.

### Reverse phase protein array (RPPA).

Cells were plated in duplicate in a 6-well plate, washed 2 times in PBS, and lysed in RPPA lysis buffer (1% Triton X-100, 50 mM HEPES, pH 7.4, 150 mM NaCl, 1.5 mM MgCl_2_, 1 mM EGTA, 100 mM NaF, 10 mM sodium pyrophosphate, 1 mM sodium orthovanadate, 10% glycerol, 1 mM PMSF, and protease inhibitor cocktail). Protein concentration was adjusted to 1.5 μg/μL in SDS sample buffer (40% glycerol, 8% SDS, 0.25 M Tris-HCl, pH 6.8, and 1.43 M 2-mercaptoethanol). The samples were sent to MD Anderson Cancer Center Core Facility for RPPA analysis. Briefly, cell lysates were serially diluted 2-fold for 5 dilutions (from undiluted to 1:16 dilution) and arrayed on nitrocellulose-coated slides in an 11 × 11 format. Samples were probed with antibodies using a tyramide-based signal amplification approach and visualized by DAB colorimetric reaction. Slides were scanned on a flatbed scanner to produce 16-bit tiff images. Spots from tiff images were identified and the density was quantified by Array-Pro Analyzer. Normalized log_2_ values were median-centered and used for heatmap generation using GraphPad Prism v7.

### Mice.

C57BL/6J (B6; stock 000664), B6.CB17-*Prkdc^scid^*/SzJ (SCID; stock 001913), B6.129S2-*Cd4^tm1Mak^*/J (CD4KO; stock 002663), and B6.129S2-*Cd8a^tm1Mak^*/J (CD8KO; stock 002665) mice were purchased from Jackson Laboratories. All animals were in the B6 genetic background. Mice used for orthotopic implantation and adoptive transfer recipients were males 8 to 10 weeks of age.

### Orthotopic implantation and tumor imaging.

Cells were trypsinized and washed twice in PBS. Mice were anesthetized with a mixture of 100 mg/kg ketamine and 10 mg/kg xylazine. The abdomen was shaved and swabbed with a sterile alcohol pad followed by povidone-iodide scrub. A small vertical incision was made over the left lateral abdominal area, to the left of the spleen. The head of the pancreas attached to the duodenum was located. Using a sterile Hamilton syringe with a 27 gauge needle, 0.5 million cells in 30 μL PBS were injected into the head of the pancreas. The injection site was pressed with a sterile cotton swab to prevent leakage. The abdominal and skin incisions were closed with 5-0 silk black-braided sutures. The mice were given an intraperitoneal injection of 2 mg/kg ketorolac immediately after surgery. To monitor tumor growth, the animals were injected intraperitoneally with 100 mg/kg RediJect D-Luciferin (PerkinElmer) and imaged on the IVIS Lumina III imaging system (Xenogen). Data were analyzed using Living Image v4.3.1 software.

### Survival studies.

Mice implanted with tumor cells were monitored by IVIS imaging every week for tumor growth. Death, weight loss of 15% or more of body weight, or inability to move were considered endpoints at which surviving animals were euthanized.

### T cell depletion using neutralizing antibodies.

B6 mice were injected intraperitoneally with 500 μg neutralizing antibodies against CD4 (BioXCell BE0003-1, clone GK1.5) and CD8 (BioXCell BE0223, clone 53-5.8) on days 1 and 3. On day 3, depletion of CD4^+^ and CD8^+^ T cells was confirmed by flow cytometry using different anti-CD4 (BioLegend 116025, clone RM4-4) and anti-CD8 (BioLegend 126610, clone YTS156.7.7) antibodies. The control mouse received PBS injections. On day 4, mice were implanted with 0.5 million αKO cells in the head of the pancreas as described above. Following implantation, mice received weekly injections of neutralizing antibodies or PBS until the end of the study. Tumor growth was monitored by IVIS imaging.

### Adoptive T cell transfer.

Six months after B6 mice were implanted with αKO cells, the mice were euthanized and their spleens collected under sterile conditions in PBS. The spleen was forced through a 70-μm filter and washed with 10 mL PBS. RBCs were lysed using ACK lysis buffer (150 mM NH_4_Cl, 10 mM KHCO_3_, and 0.1 mM Na_2_EDTA, pH 7.2–7.4). Cells were washed 2 times in PBS and collected by centrifugation at 330*g* for 5 minutes at 4°C. Splenocytes were counted and resuspended in MACS buffer for T cell isolation using the Mouse Pan T Cell Isolation Kit II (Miltenyi 130-095-130) following the manufacturer’s instructions. Cells were analyzed for CD90.2 expression by flow cytometry after purification to ensure at least 98% purity. Purified T cells from 2 or more mice were pooled and the desired number of cells was suspended in 100 μL PBS and injected retro-orbitally into SCID mice.

### Flow cytometry.

Cells were detached from the tissue culture plate using trypsin-EDTA (Corning) or StemPro Accutase (CD80 and H-2K^b^ staining). They were washed 2 times in PBS and incubated with 1:50 FcR block for 20 minutes prior to staining for 30 minutes with antibodies. Cells were washed twice in PBS and immediately analyzed by flow cytometry on a FACScalibur (BD Biosciences) or DxP 8 (Cytek). Cells to be analyzed at a later time were fixed in 2% paraformaldehyde. Data were analyzed using FlowJo.

### Histology.

Mouse organs were fixed in 10% formalin for 24 hours. Tissues were processed using a Leica ASP300S Tissue Processor, paraffin embedded, and cut into 4-μm sections. H&E staining was performed using Hematoxylin Solution, Gill No. 3 (Sigma-Aldrich) and Eosin Y (Thermo Fisher Scientific). Immunohistochemistry (IHC) was performed manually following deparaffinization and rehydration. Antigen retrieval was done in citrate buffer pH 6.0 (Vector Laboratories) using a Decloaking Chamber (Biocare Medical). Endogenous peroxidase activity and biotin were blocked using H_2_O_2_ and the Avidin/Biotin Blocking Kit (Vector Laboratories), respectively. Following incubation with primary antibodies, the R.T.U. Vectastain Kit (Vector Laboratories) and DAB in chromogen solution (Dako) were used to develop the signal. The sections were then counterstained with hematoxylin (Dako). For quantification of T cells, ImageJ was used to count cells in eight ×20 microscopic fields per mouse.

### Antibodies.

For Western blotting, PIK3CA (catalog 4249S), pAKT T308 (catalog 9275S), pAKT S473(catalog 4051S), and β-actin (catalog 4970S) were purchased from Cell Signaling Technology. Also used were AKT (Santa Cruz, sc-8312), β-tubulin (Sigma-Aldrich, T4026), and EGFR (Epitomics, 1902-1). Anti-mouse IgG secondary antibody HRP (catalog 62-6520) and anti-rabbit IgG secondary antibody HRP (catalog 31460) were from Thermo Fisher Scientific. For flow cytometry, we used anti-mouse FcR block (catalog 101302), CD90.2 (clone 30-H12), anti-mouse CD80 (clone 16-10A1), H-2K^b^ (clone AF6-88.5), Human Trustain FcX (catalog 422301), and anti-human CD80 (clone 2D10) from BioLegend, and HLA-ABC (eBioscience, clone W6/32) and annexin V (Invitrogen, catalog A23204). For histology analyses, we used F4/80 (catalog ab111101), CD3 (catalog ab16669), and CD4 (catalog ab183685) from Abcam, and CD8 (catalog 98941) from Cell Signaling Technology.

### Inhibitor studies.

KPC cells (200,000) or human pancreatic cancer cells (0.5 million) were plated per well in a 6-well plate. A quantity of 10 μM Akt inhibitor VIII (EMD Millipore, 124017) was added to cells the following day in fresh media. For the dose-response curve, cells were treated with increasing concentrations of Akt inhibitor or DMSO. Cells were incubated at 37°C and 5% CO_2_ for 48 hours. Cells were detached from the plate using StemPro Accutase, washed 2 times in PBS, incubated with FcR block, stained with anti-CD80 and anti–H-2K^b^ (mouse) or anti–HLA-ABC and anti-CD80 (human) antibodies, and analyzed by flow cytometry (FACScalibur or DxP 8). Cells from a duplicate plate were lysed and subjected to Western blotting.

### Quantitative real-time PCR (qRT-PCR).

RNA was extracted from cells using the RNeasy Kit (Qiagen). The RNase-free DNase Set (Qiagen) was used for on-column DNase digestion. cDNA was synthesized using the iScript cDNA Synthesis Kit (Bio-Rad). A StepOnePlus Real-Time PCR system was used for qRT-PCR. All reactions were carried out in triplicate using TaqMan gene expression assays. *Hprt* (Mm00446068_m1), *B2m* (Mm00437762_m1), *CD80* (Mm00711660_m1), and *H-2K^b^* (Mm01612247_mH2-K1) were purchased from Thermo Fisher Scientific. Gene expression changes were normalized to *Hprt* and analyzed by the 2^-ΔCT^ method.

### Statistics.

All statistical analyses were done using GraphPad Prism v7. For all statistical comparisons, 2-tailed Student’s *t* test was used for comparison between 2 groups. The 1-way ANOVA with Bonferroni’s post hoc test and Kruskal-Wallis test with Dunn’s post hoc test were used for multi-group comparisons. *P* less than 0.05 was considered statistically significant.

### Study approval.

All experiments in mice were conducted in accordance with the Office of Laboratory Animal Welfare and approved by the IACUC of Stony Brook University, Stony Brook, New York.

## Author contributions

NS designed and carried out experiments, analyzed and interpreted the data, and wrote the manuscript. PAM designed experiments and helped with adoptive transfers and flow cytometry experiments and analysis. HVH helped with in vitro inhibitor experiments. OP performed TCGA analysis. YPJ performed all animal surgeries. LMB helped design experiments, analyze and interpret data, and write the manuscript. KP and CL provided human pancreatic cancer cell lines and helped with data interpretation. AWMV helped with experimental design, analysis, interpretation of data, and manuscript writing. RZL supervised the project, designed experiments, analyzed and interpreted data, and helped write the manuscript.

## Supplementary Material

Supplemental data

## Figures and Tables

**Table 1:**
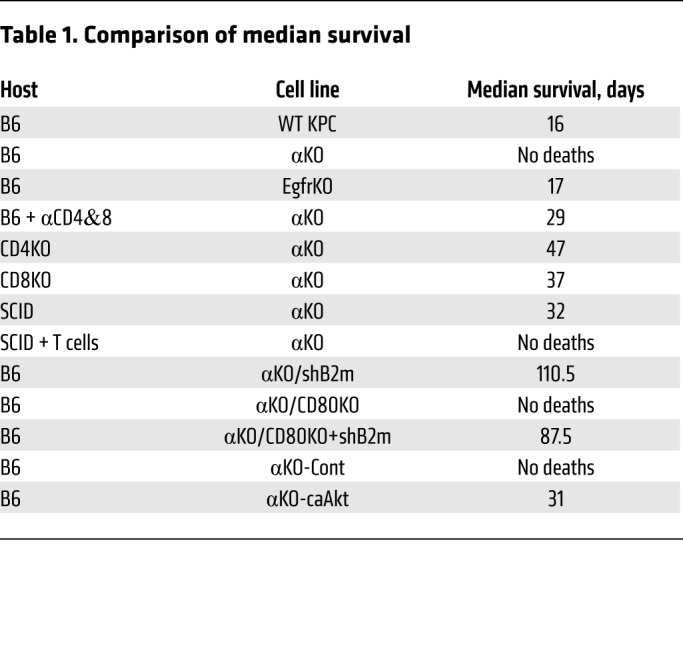
Comparison of median survival

## Version 1 05/21/2019

In-Press Preview

## Version 2 07/15/2019

Electronic publication

## Version 3 08/01/2019

Print issue publication
