# Supplementary material for: Tumor-intrinsic PIK3CA represses tumor immunogenicity in a model of pancreatic cancer
Source: J Clin Invest. 2019 Jul 15;129(8):3264–76. doi: 10.1172/JCI123540 (PMC6668699; doi:10.1172/JCI123540)
Supplement: Supplemental data [file jci-129-123540-s177.pdf]

SUPPLEMENTARY INFORMATION

Tumor-intrinsic PIK3CA Represses Tumor Immunogenecity in a Model of Pancreatic Cancer

Running title: Pik3ca and pancreatic cancer immune evasion

Nithya Sivaram[1,2], Patrick A. McLaughlin[3], Han V. Han[1,4], Oleksi Petrenko[3], Ya-Ping Jiang[1], Lisa M. Ballou[1], Kien Pham[5], Chen Liu[5], Adrianus W.M. van der Velden[3], and Richard Z. Lin[1,6]

[1]Department of Physiology and Biophysics, Stony Brook University, Stony Brook, N.Y.

[2]Molecular and Cellular Biology Graduate Program, Stony Brook University, Stony Brook, N.Y.

[3]Department of Molecular Genetics and Microbiology and Center for Infectious Diseases, Stony Brook University, Stony Brook, N.Y.

[4]Biomedical Engineering Graduate Program, Stony Brook University, Stony Brook, N.Y.

[5]Department of Pathology and Laboratory Medicine, New Jersey Medical School and Robert Wood Johnson Medical School, Rutgers University School of Medicine, Newark, N.J.

[6]Medical Service, Northport VA Medical Center, Northport, N.Y.

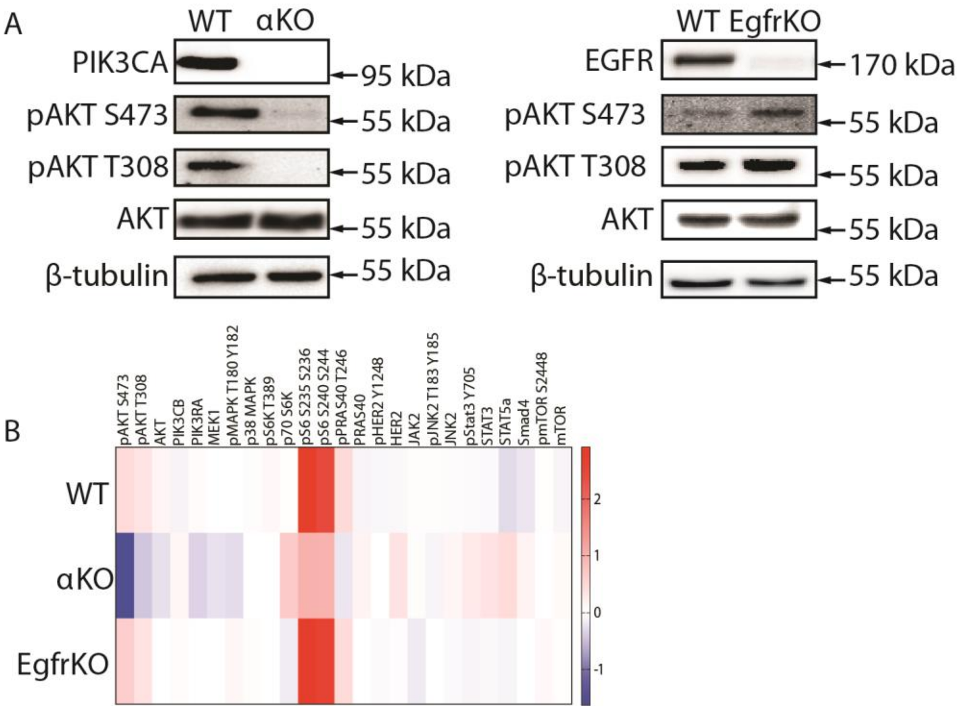

Supplementary Fig. S1. Western blotting and RPPA analysis of KPC cell lines. *Pik3ca* or *Egfr* were targeted in wildtype (WT) KPC cells using CRISPR/Cas9 and clonal cell lines were established. **(A)** Representative western blots to confirm gene ablation and to assess AKT activation. β tubulin is a loading control. Experiments were repeated 3 times. **(B)** Heatmap for RPPA analysis of the three cell lines.

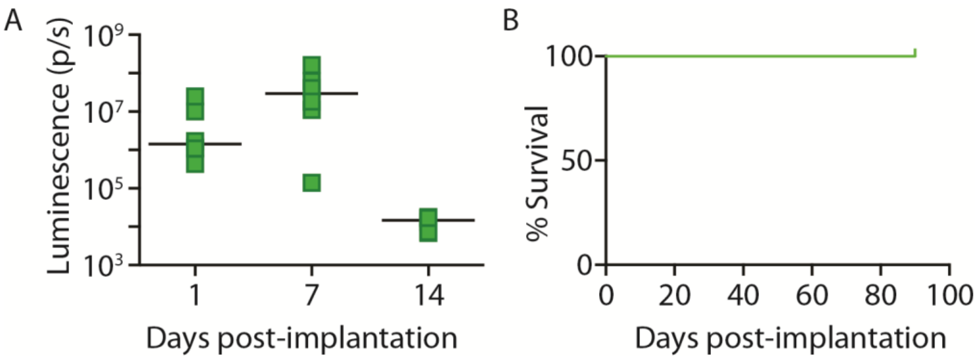

Supplementary Fig. S2. Regression of αKO clone2 tumors *in vivo*. αKO clone2 cells (0.5 million) were implanted in the head of the pancreas of B6 mice (*n* = 6) and tumor growth was monitored by IVIS imaging of the luciferase signal. **(A)** Graph shows quantification of luciferase signals. **(B)** Kaplan-Meier survival curve.

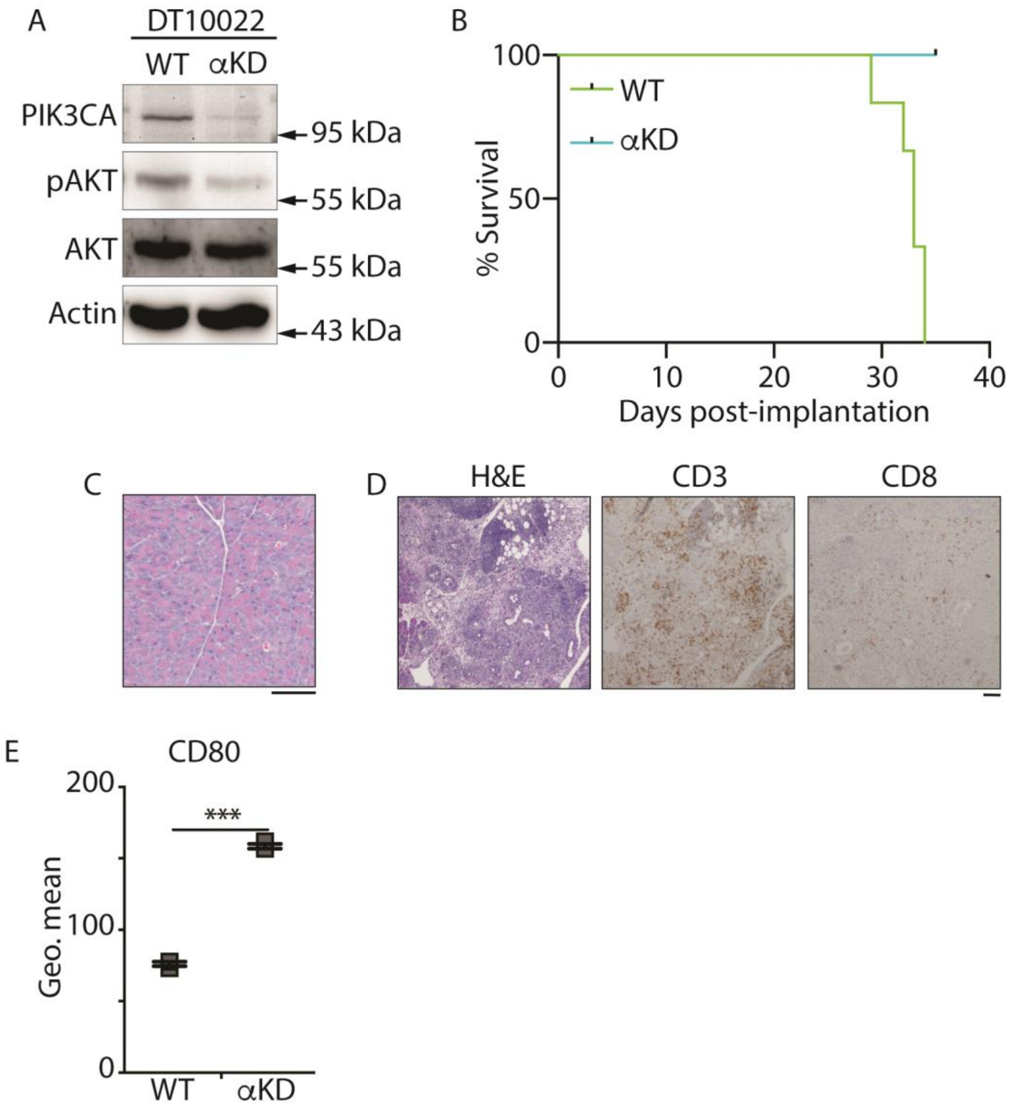

Supplementary Fig. S3. ***In vitro*** **and** ***in vivo*** **characterization of αKD cells.** *Pik3ca* was targeted in DT10022 cells using CRISPR/Cas9, and clonal cell line αKD was established. **(A)** Western blotting analysis shows a reduction in PIK3CA and pAKT levels in αKD cells as compared to WT DT10022 cells. Actin is a loading control. **(B)** Kaplan-Meier survival curves for B6 mice implanted with WT or αKD cells (0.5 million) in the head of the pancreas (n = 6). P = 0.0008 (log-rank test). **(C)** Mice implanted with αKD cells in **B** were euthanized 35 days after implantation. H&E-stained pancreatic section from a mouse with no tumor. Scale bar, 100 µm. **(D)** 0.5 million αKD cells were implanted in the head of the pancreas of B6 mice and pancreata were harvested 10 days later. Sections were stained with H&E, or IHC was performed with the indicated antibodies. Representative sections are shown. n = 3. Scale bar, 100 µm. **(E)** Cell surface CD80 levels in WT and αKD cells as determined by flow cytometry. The mean ± SEM of the geometric means (Geo. Mean) of each flow cytometry distribution is shown (n = 3). ***P = 0.0004 (paired t-test).

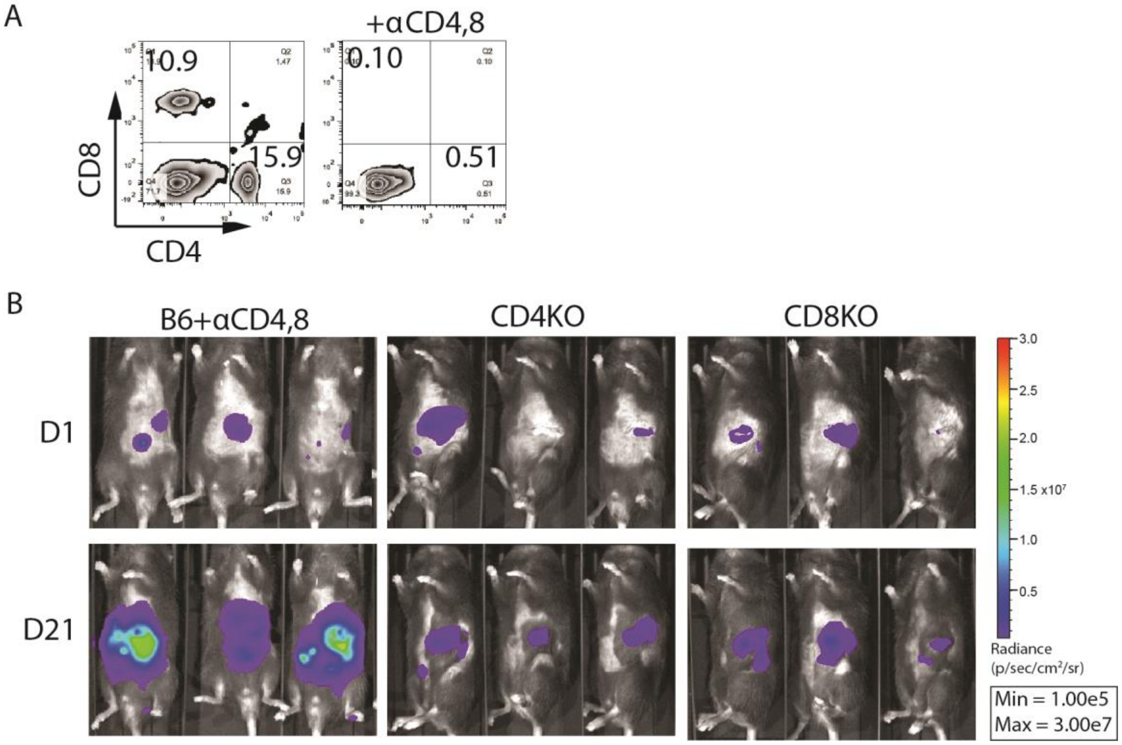

Supplementary Fig. S4. Tumor growth in mice lacking CD4 and/or CD8 T cells. (A) B6 mice were injected with neutralizing CD4 and CD8 antibodies. Peripheral blood was analyzed by flow cytometry to detect depletion of $CD4^+$ and $CD8^+$ T cells. Dot plots show $CD4^+$ and $CD8^+$ T cells in mice injected with saline (*left*) or CD4/8 antibodies (*right*). **(B)** IVIS images of B6 mice injected with CD4/8 antibodies, CD4KO mice, and CD8KO mice implanted with 0.5 million αKO cells in the head of the pancreas.

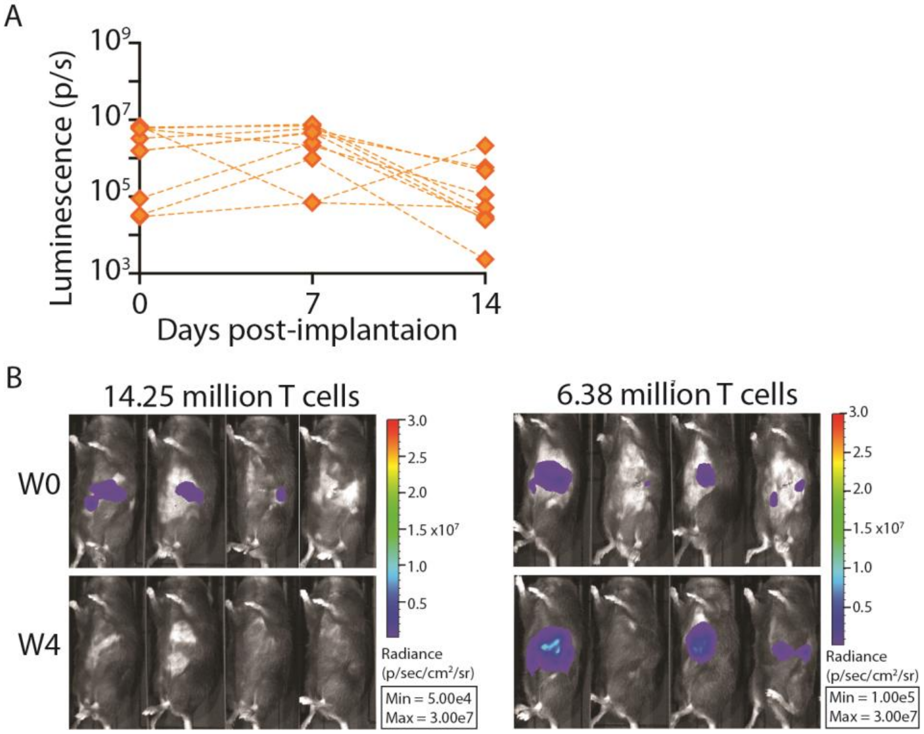

Supplementary Fig. S5. αKO tumor regression in SCID mice treated with T cells. (A) Mice were implanted with 0.5 million αKO KPC cells 24 hours after adoptive transfer of T cells from a convalescent B6 mouse previously implanted with αKO cells. Quantification of luciferase signals from each mouse. *n* = 8. **(B)** 0.5 million αKO KPC cells were implanted in the head of the pancreas of SCID mice (*n* = 8). Four days later, the mice were imaged (t = W0) and then treated with 14.25 million or 6.38 million T cells (*n* = 4 per group) from a convalescent B6 mouse previously implanted with αKO cells. IVIS images show luciferase signals as a measure of tumor volume before and 4 weeks after treatment.

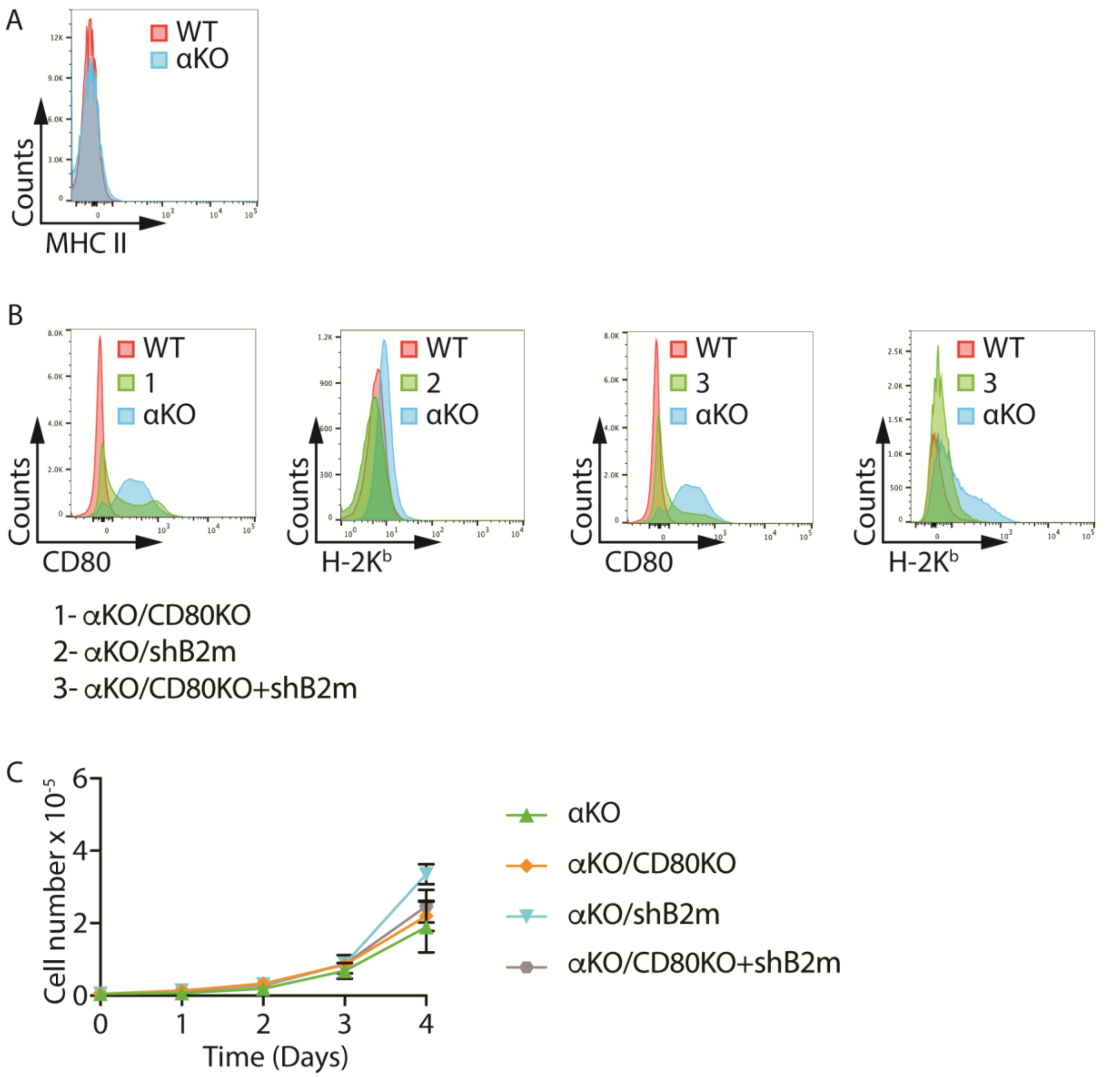

Supplementary Fig. S6. Cell surface levels of MHC II, H-2K^{b}, and CD80 in KPC cell lines. Flow cytometric analysis to determine levels of **(A)** MHC II (I-A^{b}) and **(B)** H-2K^{b} and CD80. **(C)** Proliferation rates of αKO KPC cell lines in standard 2D culture. Cells plated in triplicate were counted at the times indicated (mean ± SEM; n = 3). The three cell lines grew at rates similar to αKO cells (One-way ANOVA with Bonferroni's multiple comparison's test).

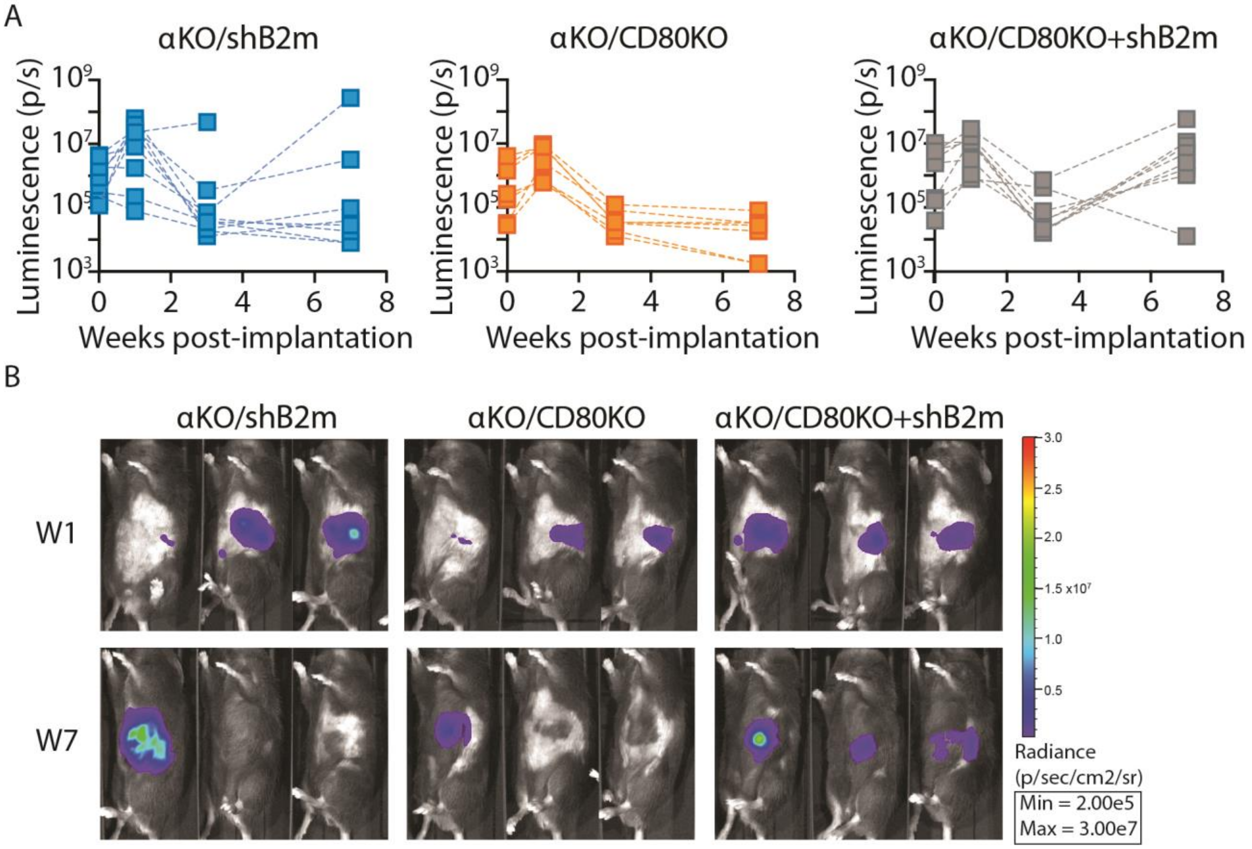

Supplementary Fig. S7. *In vivo* tumor growth. B6 mice were implanted with 0.5 million αKO/shB2m, αKO/CD80KO, or αKO/CD80KO+shB2m cells in the head of the pancreas, and tumor growth was monitored by IVIS imaging. **(A)** Quantification of the luciferase signals in each mouse. αKO/shB2m, *n* = 8; αKO/CD80KO, *n* = 7; αKO/CD80KO+shB2m, *n* = 8. **(B)** Representative images of 3 mice in each group are shown.

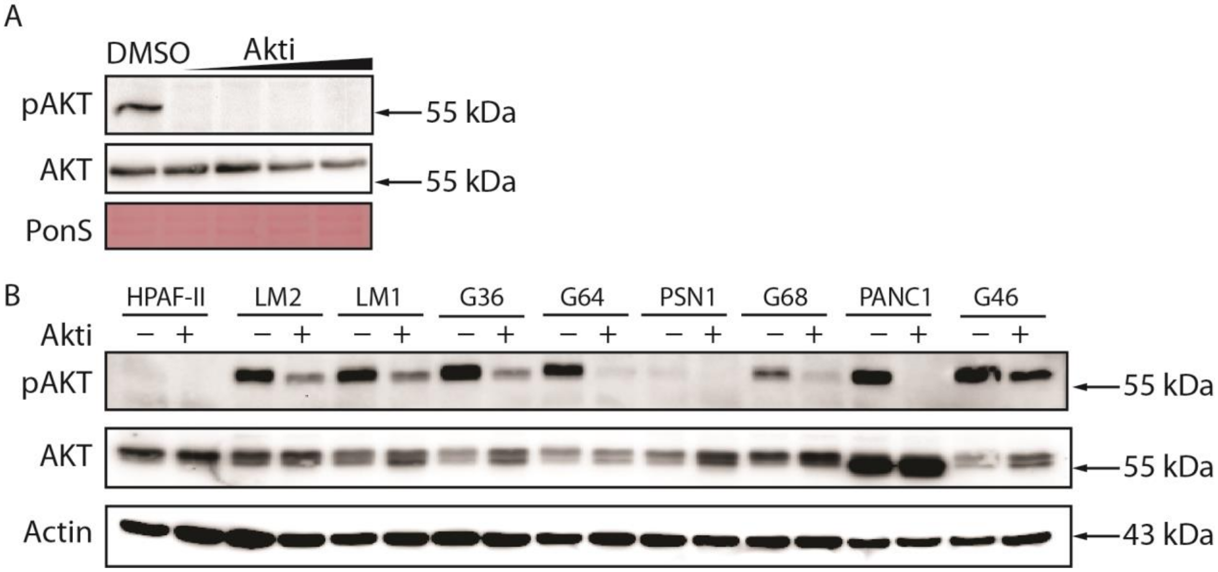

Supplementary Fig. S8. Effect of Akti treatment on phospho-AKT. (A) WT KPC cells were treated with increasing concentrations of Akti or **(B)** human PDAC cell lines were treated with 10 μM Akti for 48 hours. Cells were lysed in RIPA buffer and total protein was run on a denaturing gel. Western blots show levels of phospho- and total AKT. Actin in a loading control. PonS, Ponceau S-stained blot.

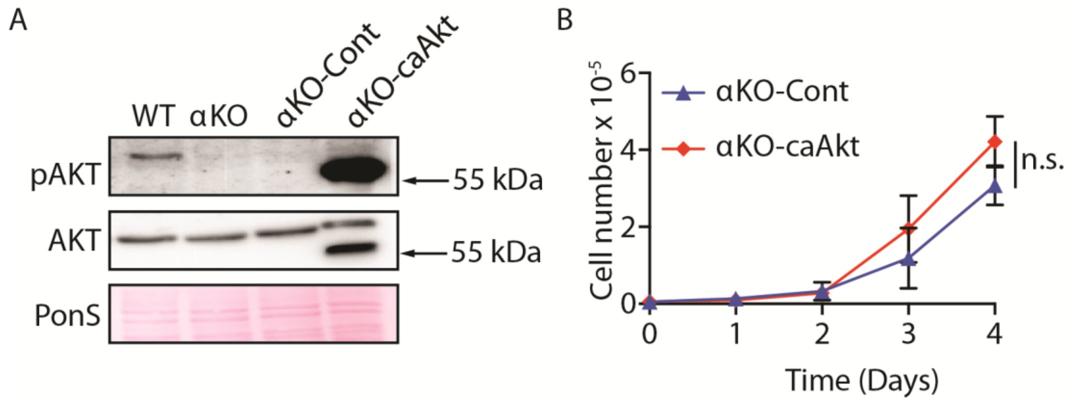

Supplementary Fig. S9. AKT phosphorylation and *in vitro* growth of αKO-caAkt cells. (A) αKO cells were infected with lentivirus expressing control vector (Cont) or caAkt. Western blots confirm the expression of caAkt. PonS, Ponceau S-stained blot. **(B)** Proliferation rates in standard 2D culture. Cells plated in triplicate were counted at the times indicated (mean ± SEM; n = 3). n.s., not significant.

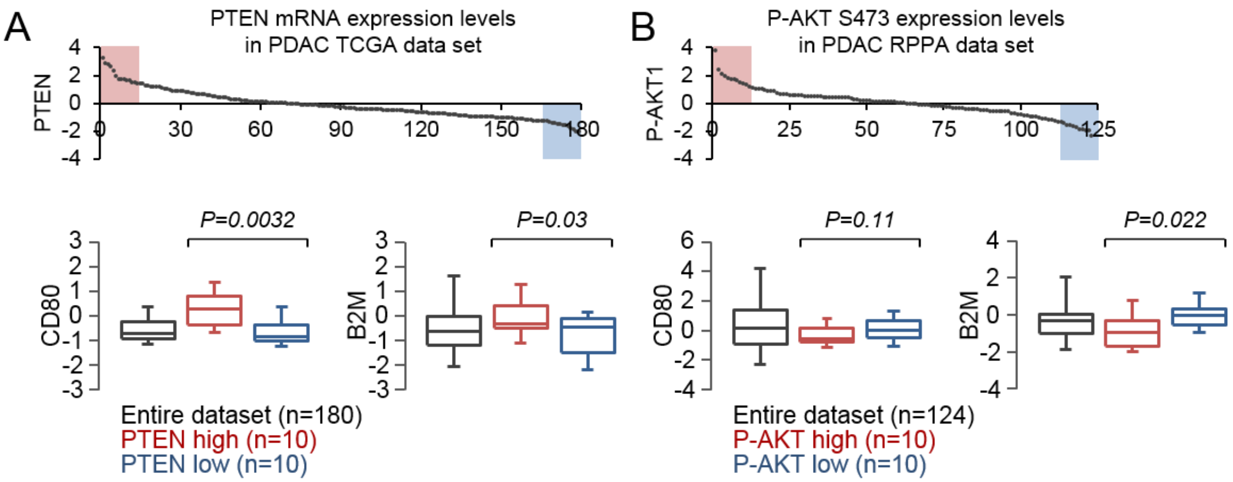

Supplementary Fig. S10. Correlation between PIK3CA/AKT signaling and expression of CD80 and B2M in PDAC patients. The Cancer Genome Atlas (TCGA) data were downloaded as z-scores from the cBioPortal (http://www.cbioportal.org). RNAseqV2 data were available for PTEN, CD80 and B2M. The RPPA data contain phospho-AKT1 levels. **(A)** *Top*, range of PTEN mRNA expression levels in the PDAC data set. Shading shows 10 samples with high (pink) or low (blue) PTEN expression. *Bottom*, box plots comparing CD80 and B2M mRNA levels in patients with high or low PTEN expression (Student's *t*-test). **(B)** *Top*, range of phospho-AKT1 S473 expression levels in the PDAC data set. Shading shows 10 samples with high (pink) or low (blue) phospho-AKT expression. *Bottom*, box plots comparing CD80 and B2M mRNA levels in patients with high or low phospho-AKT levels (Student's *t*-test).

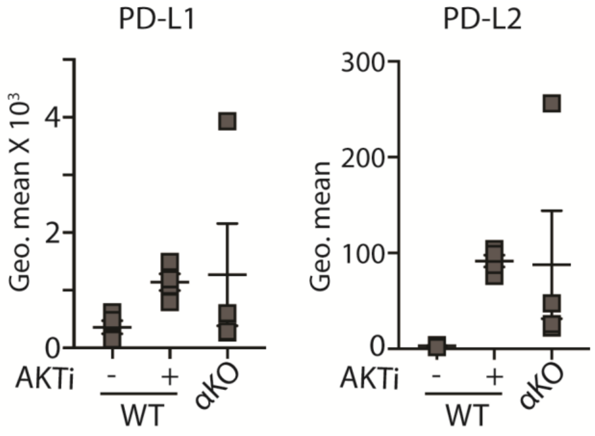

Supplementary Fig. S11. Cell surface levels of PD-L1 and PD-L2 in WT and αKO cells. WT KPC cells were treated with 10 μM Akti for 48 hours. Cell surface levels of PD-L1 and PD-L2 were quantified by flow cytometry. WT cells treated with DMSO and αKO cells were used for comparison. The graph shows the mean ± SEM of the geometric means (Geo. Mean) of each flow cytometry distribution. n = 4. The data did not reach statistical significance (One-way ANOVA with Bonferroni's post hoc test).

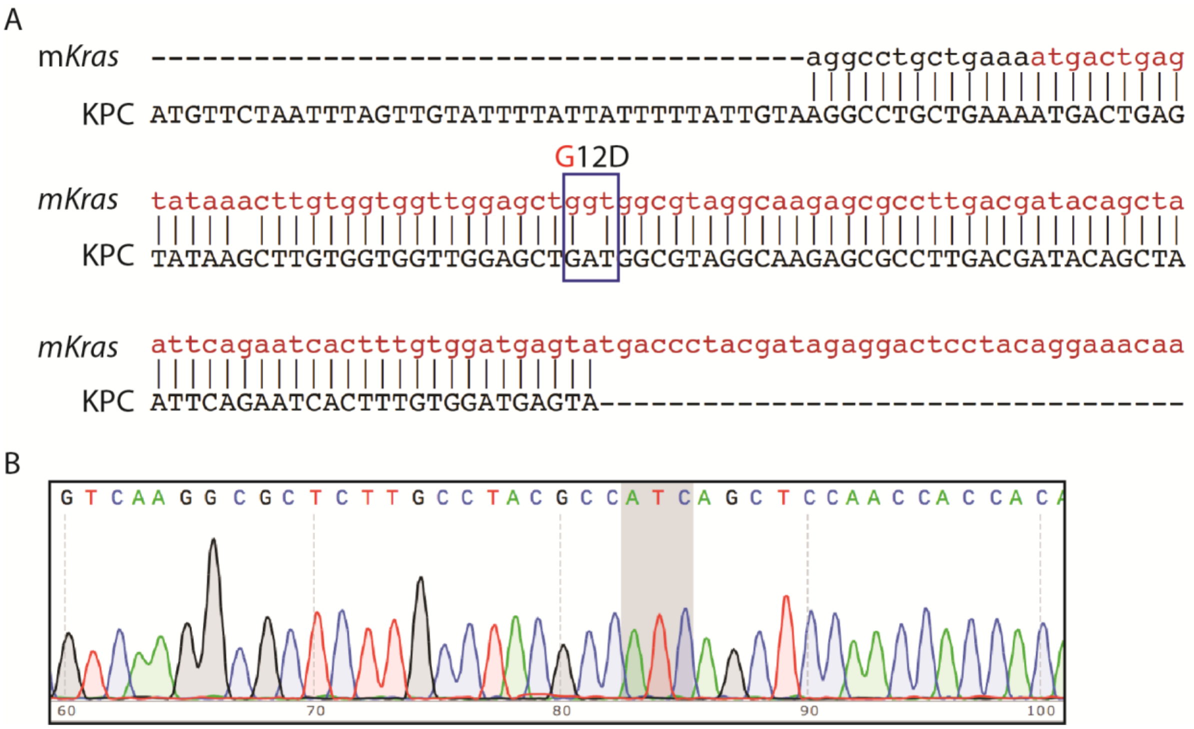

Supplementary Fig. S12. Sequence of *Kras* exon 1. Genomic DNA from WT KPC cells was used for sequencing exon 1 of the *Kras* gene. **(A)** Sequence alignment of DNA from KPC cells (KPC) against the murine *Kras* gene (NCBI Gene ID 16653) (m*Kras*). **(B)** Chromatogram showing G12D mutation (grey highlight) in WT KPC cells.
